# Radiotherapy Suppresses Angiogenesis in Mice through TGF-βRI/ALK5-Dependent Inhibition of Endothelial Cell Sprouting

**DOI:** 10.1371/journal.pone.0011084

**Published:** 2010-06-11

**Authors:** Natsuko Imaizumi, Yan Monnier, Monika Hegi, René-Olivier Mirimanoff, Curzio Rüegg

**Affiliations:** 1 Division of Experimental Oncology, Centre Pluridisciplinaire d'Oncologie (CePO), Centre Hospitalier Universitaire Vaudois (CHUV) and University of Lausanne (UNIL), Epalinges, Switzerland; 2 NCCR Molecular Oncology, ISREC-EPFL, Epalinges, Switzerland; 3 Laboratory of Brain Tumor Biology and Genetics, Centre Universitaire Romand de Neurochirurgie, Centre Hospitalier Universitaire Vaudois (CHUV) and University of Lausanne (UNIL), Lausanne, Switzerland; 4 Department of Radio-Oncology, Centre Hospitalier Universitaire Vaudois (CHUV) and University of Lausanne (UNIL), Lausanne, Switzerland; 5 Department of Medicine, Faculty of Science, University of Fribourg (UNIFR), Fribourg, Switzerland; Wayne State University School of Medicine, United States of America

## Abstract

**Background:**

Radiotherapy is widely used to treat cancer. While rapidly dividing cancer cells are naturally considered the main target of radiotherapy, emerging evidence indicates that radiotherapy also affects endothelial cell functions, and possibly also their angiogenic capacity. In spite of its clinical relevance, such putative anti-angiogenic effect of radiotherapy has not been thoroughly characterized. We have investigated the effect of ionizing radiation on angiogenesis using *in vivo, ex vivo* and *in vitro* experimental models in combination with genetic and pharmacological interventions.

**Principal Findings:**

Here we show that high doses ionizing radiation locally suppressed VEGF- and FGF-2-induced Matrigel plug angiogenesis in mice *in vivo* and prevented endothelial cell sprouting from mouse aortic rings following *in vivo* or ex *vivo* irradiation. Quiescent human endothelial cells exposed to ionizing radiation *in vitro* resisted apoptosis, demonstrated reduced sprouting, migration and proliferation capacities, showed enhanced adhesion to matrix proteins, and underwent premature senescence. Irradiation induced the expression of P53 and P21 proteins in endothelial cells, but *p53* or *p21* deficiency and P21 silencing did not prevent radiation-induced inhibition of sprouting or proliferation. Radiation induced Smad-2 phosphorylation in skin *in vivo* and in endothelial cells *in vitro*. Inhibition of the TGF-β type I receptor ALK5 rescued deficient endothelial cell sprouting and migration but not proliferation *in vitro* and restored defective Matrigel plug angiogenesis in irradiated mice *in vivo*. ALK5 inhibition, however, did not rescue deficient proliferation. Notch signaling, known to hinder angiogenesis, was activated by radiation but its inhibition, alone or in combination with ALK5 inhibition, did not rescue suppressed proliferation.

**Conclusions:**

These results demonstrate that irradiation of quiescent endothelial cells suppresses subsequent angiogenesis and that ALK5 is a critical mediator of this suppression. These results extend our understanding of radiotherapy-induced endothelial dysfunctions, relevant to both therapeutic and unwanted effects of radiotherapy.

## Introduction

Radiotherapy is a well-established therapeutic modality in clinical oncology. It is applied to over half of all cancer patients during the course of their disease, as curative-intent, adjuvant or palliative treatment [1]. The main mechanism by which radiotherapy is thought to exert its therapeutic anti-cancer effects is through the induction of double-strand DNA breaks. The extent of the DNA damage in cancer cells together with their proliferative and the genetic conditions, will determine whether irradiated cancer cells will survive, will undergo senescence or will die by apoptosis or mitotic catastrophe [2]. Radiotherapy also affects non-tumoral cells present in the tumor microenvironment and surrounding tissues, including endothelial cells [3]. Ionizing radiation was reported to induce apoptosis of endothelial cells of the tumor vasculature, resulting in tumor vessel disruption and delayed tumor growth [4]. Moreover, radiotherapy can affect quiescent endothelium in healthy tissues. Microvessels (capillaries and sinusoids) are most sensitive, displaying damages of endothelial cells, resulting in teleangectasia, capillary rupture and thrombosis. Medium-size vessels show neointimal proliferation, fibrinoid necrosis, thrombosis and acute arteritis. Large vessels are less affected, although arterial thrombosis and atheromatosis were reported [5]. These radiation-induced vascular modifications are associated with increased risk of serious complications in radiotherapy-treated patients, including arterial occlusions, heart attacks and stroke [6], [7], [8]. Furthermore, it has been supposed that exposure of quiescent vasculature to ionizing radiation might inhibit a subsequent angiogenic response. Endothelial dysfunctions and impaired angiogenesis are thought to contribute to late tissue damages observed after radiotherapy including perturbed wound healing, tissue fibrosis and organ dysfunctions [9].

In spite of its clinical relevance, whether irradiation of quiescent vasculature impinges on subsequent (*de novo*) angiogenesis and the possible mechanisms involved have not been thoroughly investigated. In this work we have addressed these questions through *in vivo* and *in vitro* experiments in combination with genetic and pharmacological interventions. Here we report that irradiation prevents vascular growth factor (VEGF) and fibroblast growth factor-2 (FGF-2) -induced angiogenesis *in vivo*, and suppresses endothelial cell proliferation, migration and sprouting *in vitro*. Radiation activates the TGF-β type I receptor/activin receptor-like kinase-5 (ALK5) pathway and pharmacological ALK5 inhibition prevents radiation-induced suppression of endothelial cell migration and sprouting *in vitro*, and impairment of angiogenesis *in vivo*. By demonstrating that irradiation of quiescent vasculature inhibits subsequent angiogenic responses and by providing a cellular and molecular mechanism, these results broaden our understanding of radiation-induced endothelial cell dysfunctions relevant to normal tissue homeostasis and cancer biology.

## Results

### High-dose ionizing radiation inhibits VEGF and FGF-2-induced angiogenesis in healthy skin

**Figure 1:**
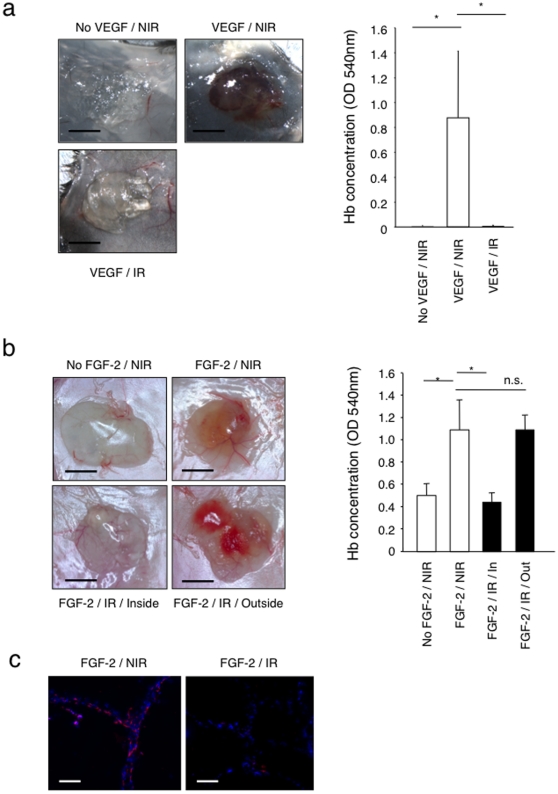
Inhibition of Matrigel plug angiogenesis by skin pre-irradiation. (a) VEGF-induced Matrigel plug angiogenesis assay was performed within non-irradiated or 20 Gy pre-irradiated areas on the back of distinct C57/BL6 mice. Bar = 0.5 cm. *P<0.05. (n = 7). (b) FGF-2 induced Matrigel plug angiogenesis assay was performed within non-irradiated area either on the back of distinct Swiss nude mice (upper panels) or within and outside a 20 Gy irradiated dorsal area on the same mouse (lower panes) (n = 7). Matrigel without growth factor was used as a negative control. Angiogenesis was quantified by measuring haemoglobin concentration in the recovered Matrigel plugs. Bar = 0.5 cm. *P<0.0001. (c) Staining of CD31 positive endothelial cells (red) and DAPI (blue) was performed on frozen Matrigel sections. Bar = 60 µm. Matrigel implanted within the irradiated area lacks endothelial cells (n = 10). NIR: non-irradiated, IR: irradiated.

To test whether high doses of ionizing radiation might inhibit *de novo* angiogenesis, we performed Matrigel plug angiogenesis assays [10] in non-irradiated mice and in locally pre-irradiated mice (single X-ray dose of 20 Gy at the site of plug implantation). This dose corresponds to a biological cumulative dose of 50–60 Gy (based on the linear-quadratic model depending on the chosen α/β values) delivered to patients during fractionated radiotherapy, and is therefore of clinical significance [11]. Tissue pre-irradiation fully suppressed vascular endothelial growth factor (VEGF) - and fibroblast growth factor-2 (FGF-2) - induced angiogenesis, as determined by macroscopic examination and by measuring the haemoglobin content of the recovered plugs (Figure 1a and 1b). CD31 immunofluorescence staining of the Matrigel plugs confirmed the absence of blood vessels ingrowths into plugs implanted within the pre-irradiated tissue, compared to plugs implanted in non-irradiated tissue (Figure 1c). Also, angiogenesis occurred normally in Matrigel plugs implanted outside the pre-irradiated area in the same mice, indicating that the effect is not systemic but rather restricted to the irradiated tissue (Figure 1b, FGF-2/IR/Outside).

These results demonstrate that radiation inhibits VEGF- and FGF-2-induced *de novo* angiogenesis and that the effect is limited to the irradiated tissue.

### Ionizing radiation does not induce apoptosis in quiescent endothelial cells

**Figure 2:**
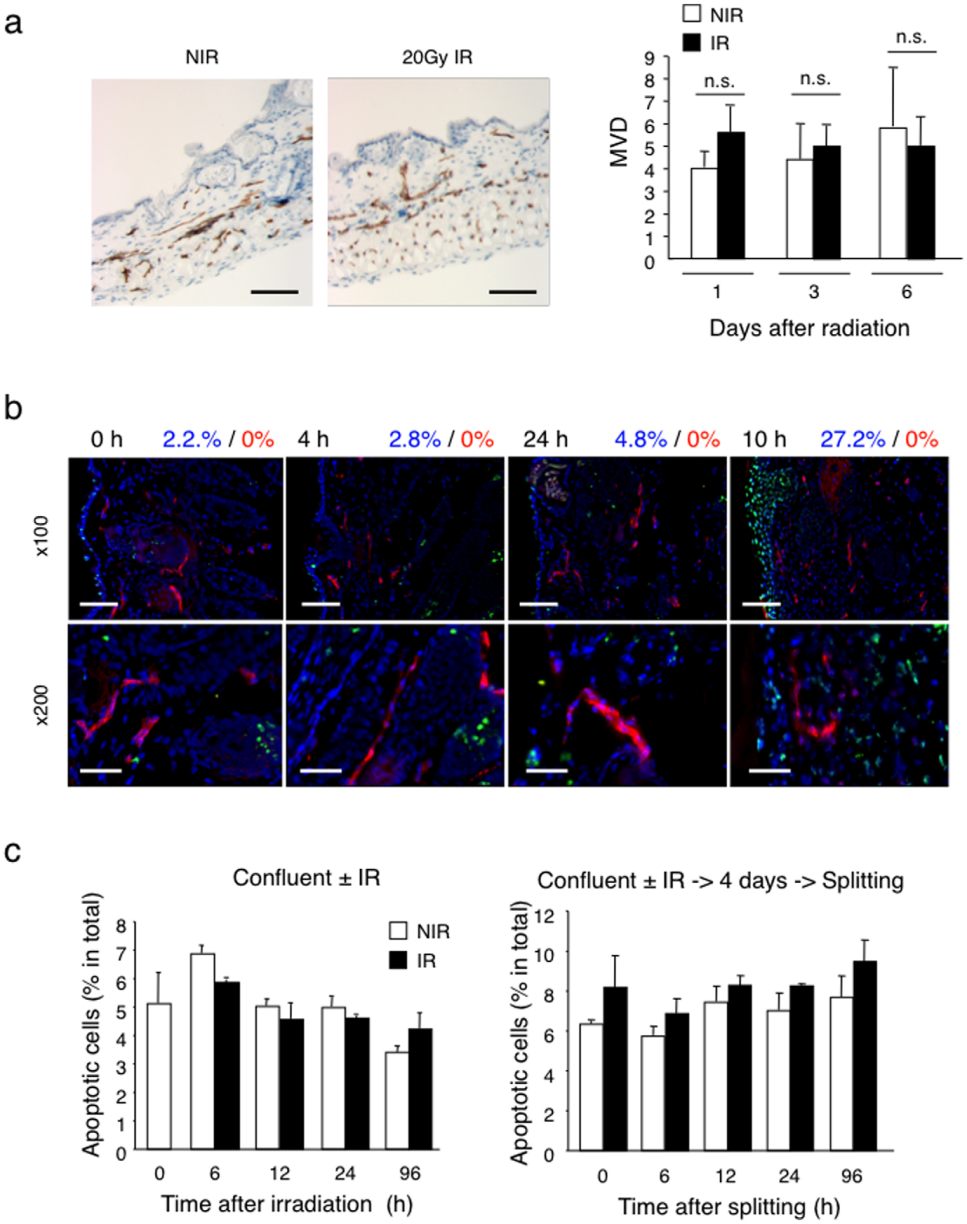
Quiescent endothelial cells *in vivo* and *in vitro* are resistant to radiation-induced apoptosis. (a) Sections of non-irradiated skin and of 20 Gy irradiated skin 6 days after irradiation were stained by immunohistochemistry for CD31 (brown). Bar = 60 µm. The graph on the right represents the microvessel density (MVD) of the skin at day 1, 3 and 6 after irradiation and non-irradiated controls. For quantification 5 regions were selected from each slide (n = 5). There was no significant difference in the MVD of irradiated *vs.* non-irradiated skin. (b) Apoptosis detection in irradiated skin by TUNEL assay. Frozen skin sections prepared before (t = 0), and 4 hours, 24 hours and 10 days after local radiation (20 Gy) were stained with TUNEL reaction (green), anti-CD31 mAb (red) and DAPI (blue). Bars  = x100: 60 µm, x200: 30 µm At 10 days after radiation, keratinocytes within the irradiated skin are largely TUNEL positive, while endothelial cells are negative. The numbers above the images give the quantification of TUNEL positive cells within the total cell population (blue) and CD31^+^ endothelial cells (red). No TUNEL^+^ endothelial cells were detected. (c) Detection of apoptosis in irradiated confluent HUVEC cultures. Left panel: frequency of apoptotic cells 4 days after 15 Gy irradiation of confluent HUVEC. Right panel: frequency of apoptotic cells in HUVEC cultures irradiated, split 4 days after radiation and cultured for another 4 days. There was no significant increase in apoptosis within irradiated confluent HUVEC cultures. Apoptotic cells were detected by Annexin V/7AAD staining. NIR: non-irradiated, IR: irradiated.

Next, we tested whether deficient angiogenesis observed in Matrigel plugs was due to radiation-induced disruption of pre-existing vessels in the irradiated area into which plugs were implanted. First we determined the microvascular density (MVD) in the skin 6 days after local irradiation (20 Gy, single dose) *vs.* no irradiation. No significant differences in vascular morphology and MVD were observed (Figure 2a). To directly assess whether radiation might induce apoptosis in quiescent endothelial cells, we performed *in situ* TUNEL assays and CD31 co-staining of skin before and at various time points after irradiation. Up to 10 days after irradiation there was no evidence for the appearance of TUNEL-positive endothelial cells in the irradiated dermis (Figure 2b). In contrast, we observed TUNEL-positive cells in the epidermis and dermis 10 days after irradiation, consistent with radiation-induced apoptosis of keratinocytes and fibroblasts [12], [13]. Moreover, we monitored the induction of apoptosis in confluent HUVEC cultures by radiation (15 Gy) using AnnexinV and 7AAD double staining. No significant loss of cells or increase in the apoptotic fraction was observed in irradiated confluent cultures maintained confluent or passaged four days after irradiation (Figure 2c, and Figure S1a). Irradiation of proliferating HUVEC (i.e. sub-confluent cultures) resulted in massive death within four days after irradiation (Figure S1b).

From these results we conclude that local irradiation does not disrupt quiescent dermal vessels *in vivo* and does not induce apoptosis in quiescent dermal endothelial cells *in vivo*, nor in confluent HUVEC *in vitro*.

### Radiation of confluent HUVEC induces premature senescence

**Figure 3:**
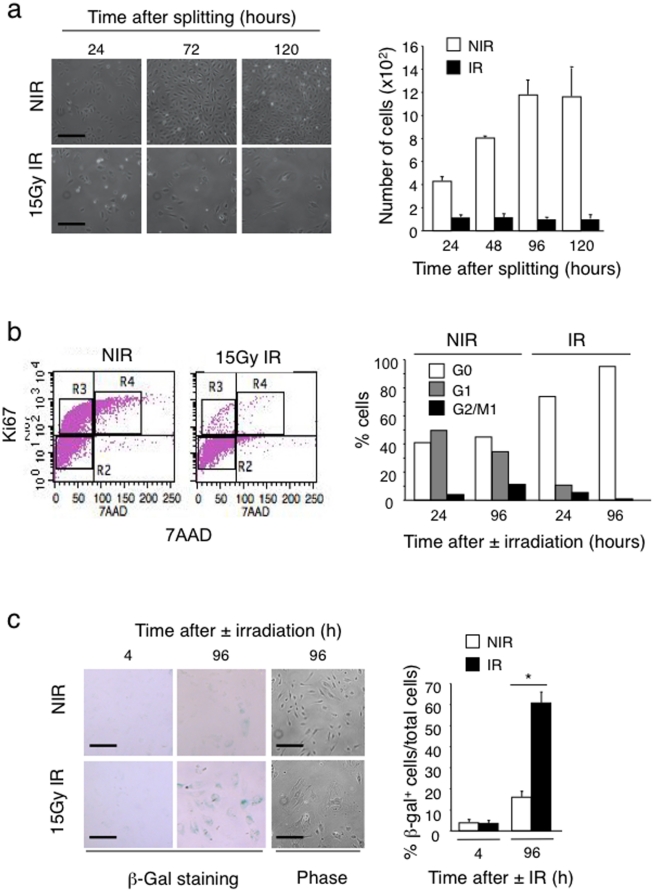
Irradiation induces senescence of confluent endothelial cells. (a) Confluent HUVEC were irradiated with 15 Gy or not, and 4 days later cultures were split at 1∶3 dilutions and cultures photographed at 1, 3, and 5 days after splitting. Right panel gives the number of cells in non-irradiated and irradiated cultures at the indicated times (n = 5). Bars = 50 µm. (b) Flow cytometry-based cell cycle analysis of confluent HUVEC collected 4 days after radiation. Cells were stained with an anti-Ki67 antibody and 7-AAD dye. Bars show the relative percentage of HUVEC in the different phases of the cell cycle (G0, G1 and G2/M1). (c) Non-irradiated and 15 Gy irradiated HUVEC were fixed 4 or 96 hours after irradiation and stained to detect senescence-associated β-galactosidase activity at pH = 6. Pictures show stained (β-gal staining) and unstained cultures (phase). Irradiated HUVEC acquired β-gal staining and a flattened, enlarged and granular cytoplasm. The bar graph gives the number of β-gal positive cells normalized by total number of cells. (n = 5). *P<0.00001.

HUVEC that were irradiated as confluent cultures and further passaged (1∶3 dilutions) remained sparse compared to non-irradiated cultures, which reached confluence four days after splitting (Figure 3a). This observation suggested that irradiation of confluent endothelial cells prevented re-entry into the cell cycle once cells were placed in proliferation-inducing conditions. We therefore analyzed the effect of irradiation on cell cycle by measuring Ki67 expression and 7AAD staining by multiparameter flow cytometry [14]. Confluent, non-irradiated HUVEC were approximately 40% in G0, 50% in G1 and less than 10% in G2/M while confluent irradiated HUVEC were over 90% in G0 and less than 10% in G1 and G2/M (Figure 3b). Cell cycle quiescence and lack of apoptosis suggested the possibility that irradiation induced endothelial cell senescence. To collect further evidence for this we performed senescence-associated β-galactosidase staining of confluent irradiated and non-irradiated HUVEC cultures. Sixty percent of irradiated HUVEC were β-galactosidase positive 4 days after irradiation compared to only 16% in control cultures. Of note, irradiated HUVEC developed a large and flattened cell shape with granular cytoplasm, another hallmark of senescent cells (Figure 3c).

Based on these results we conclude that irradiation of confluent endothelial cells prevents re-entry into the cell cycle and induces premature senescence.

### Ionizing radiation inhibits endothelial cell sprouting

**Figure 4:**
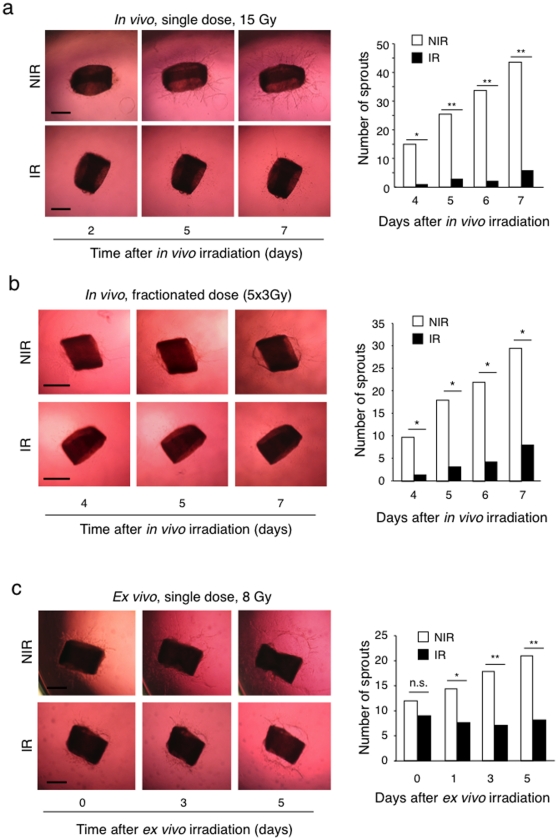
Irradiation inhibits endothelial sprouting from aortic rings. (a) C57/BL6 mice received 15 Gy whole body irradiation. Aorta were explanted at day 5, sliced into rings and embedded in collagen I gels. The number of sprouts was quantified and images were taken at the indicated days after embedding. Aortic rings obtained from non-irradiated mice were used as control. *P<0.01, **P<0.001. (b) C57/BL6 mice received 5 times 3 Gy whole body radiation every 2 days (total dose  = 15 Gy). The aorta was explanted 5 days after the last dose, sliced into rings and embedded in collagen I gels. Aortic rings obtained from non-irradiated mice were used as positive controls. The number of sprouts was quantified and images were taken at the indicated days. Bars = 500 µm. *P<0.0001. NIR: non-irradiated, IR: irradiated. (c) Aortas were explanted from wild type C57/BL6 mice, sliced into rings, embedded in collagen I gels and 2 days later irradiated with 8 Gy. The number of sprouts was quantified immediately before (t = 0), and at 1, 3, and 5 days after radiation. Bars = 500 µm. *P<0.01, **P<0.001. n.s, non significant. For each assay the number of sprouts was counted manually each day under a dissection microscope. Bar graphs on the right represent the number of sprouts. White bars: sprouting from non-irradiated mice or rings; Black bars, sprouting from irradiated mice or rings.

**Figure 5:**
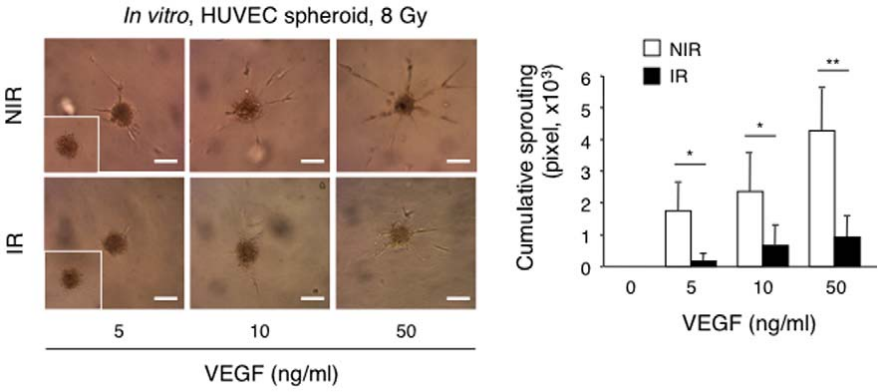
Irradiation inhibits sprouting of isolated endothelial cells. HUVEC spheroids were embedded in collagen gels and exposed to 8 Gy radiation. The cumulative sprout length was quantified from 10 randomly observed spheroids in each condition 24 hours later. There is a dose-dependent decrease of sprouting in irradiated cultures. Bars = 100 µm. *P<0.01, **P<0.001. NIR: non-irradiated, IR: irradiated.

Next we assessed whether irradiation had a direct impact on endothelial cell sprouting using the *ex vivo* aortic ring endothelial cell sprouting assay [15]. In a first experiment we exposed mice to 15 Gy, single dose, whole body irradiation and 5 days later we removed the aorta to perform the assay. Irradiation strongly suppressed VEGF-induced sprouting compared to non-irradiated control (Figure 4a). We also treated mice with fractionated radiation therapy to mimic the clinical situation in which patients are treated with multiple low doses. Radiation was given as 3 Gy single doses, five times every 2 days. Consistent with the single dose treatment experiment, we observed a significant decrease in endothelial cell sprouting also with fractionated therapy (Figure 4b). In a second experimental setting we embedded non-irradiated aortic rings in collagen gels and irradiated them two days later (8 Gy, single dose), a time at which sprouting had already started. Irradiation effectively suppressed further sprouting, without causing regression of already formed sprouts (Figure 4c). In a third experimental model we performed an endothelial cell spheroid sprouting assays using purified HUVEC [16] to exclude that cells other then endothelial cells present in the vascular wall of aortic rings, in particular, fibroblasts, could potentially influence endothelial cell sprouting in response to radiation through paracrine effects [17]. Gel-embedded HUVEC spheroids were irradiated (8 Gy, single dose) and sprouting induced by VEGF. Also in this model radiation significantly suppressed VEGF-induced HUVEC sprouting (Figure 5).

Taken together these experiments demonstrate that irradiation effectively inhibits endothelial cell sprouting though a direct effect on endothelial cells.

### Ionizing radiation suppresses endothelial cell migration

**Figure 6:**
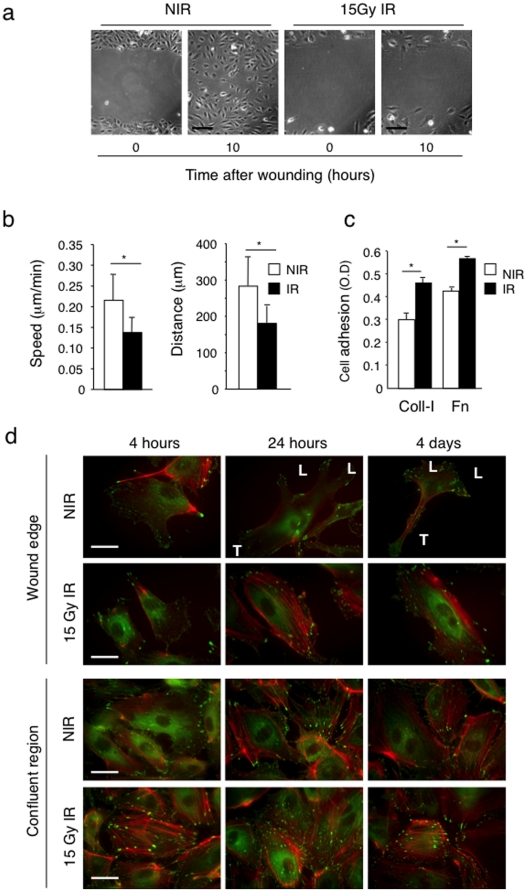
Irradiation impairs endothelial cell migration. (a) The effect of radiation on cell migration was examined by a scratch wound closure assay *in vitro*. Confluent HUVEC were irradiated with 15 Gy, and 4 days later a scratch-wound was created. The micrographs represent the wound immediately after the scratch (t = 0) and at 10 hours later. Bars = 30 µm (b) Wound closure was monitored by time-lapse microscopy and the distance and speed of individual cells were calculated. Ionizing radiations inhibit HUVEC migration. *P<0.0001. (c) Non-irradiated and 15 Gy irradiated HUVEC were tested for adhesion to collagen I and fibronectin on a short-term adhesion assay. *P<0.0001. Coll-I: collagen I, Fn: fibronectin. Irradiated cells adhere more efficiently to both substrates. (d) Radiation effect on adhesion complexes and the cytoskeleton. Confluent HUVEC were irradiated with 15 Gy. Four, 24 hours and 4 days after radiation, a line-scratch wound was made on the cell monolayer, and 10 hours later, cultures were fixed and stained by immunofluorescence with phalloidin (red) and anti-paxillin antibody (green) to reveal the actin cytoskeleton and focal adhesions. Pictures of cells at the wound edge and at confluent region are shown. Migrating NIR cells at the wounding edge are polarized (L, leading edge; T, trailing end), while non-migrating IR cells and cells in confluent regions are not polarized. Bars = 5 µm. NIR: non-irradiated, IR: irradiated conditions.

Since endothelial cell migration is an essential event for sprouting angiogenesis [18], we tested whether ionizing radiation might inhibit endothelial cell migration. To this purpose we compared the migratory capacity of irradiated (15 Gy, single dose), *vs.* non-irradiated HUVEC in a scratch wound closure assay. In non-irradiated cultures wound closure was nearly completed after 10 hours, while in irradiated cultures the wound remained largely open (Figure 6a). Single cell tracking revealed that irradiated HUVEC migrated at reduced speed, resulting in a decreased migration distance, compared to non-irradiated HUVEC (Figure 6b). Concomitantly, irradiated HUVEC displayed an increased adhesion to collagen and fibronectin substrates, as tested in a short-term adhesion assay (Figure 6c). Of interest, HUVEC undergoing replicative senescence after 20 passages (expressing senescence-associated β-galactosidase activity, data not shown) also had a decreased migration comparable to that observed in radiation-treated HUVEC (Figure S2).

Non-irradiated, migrating HUVEC at the wound margin showed a typical polarized phenotype with lamellipodia formation at the leading front, decreased actin stress fibers, appearance of cortical actin, accumulation of focal adhesions at the leading front (L) and at the trailing edge (T) (Figure 6d). Confluent HUVEC distant from the wound edge were not polarized, retained a cobble-stone-like morphology, focal adhesions around the cell periphery and well-formed actin stress fibers. Irradiated HUVEC at the wound edge showed little signs of polarization, retained a well spread morphology, focal adhesion distribution and F-actin stress fibers comparable to confluent, non-migrating cells (Figure 6d).

These results demonstrate that irradiation of quiescent endothelial cells effectively suppresses subsequent their migratory capacities.

### Radiation-induced activation of the P53-P21 pathway is not critical for the inhibition of endothelial sprouting

**Figure 7:**
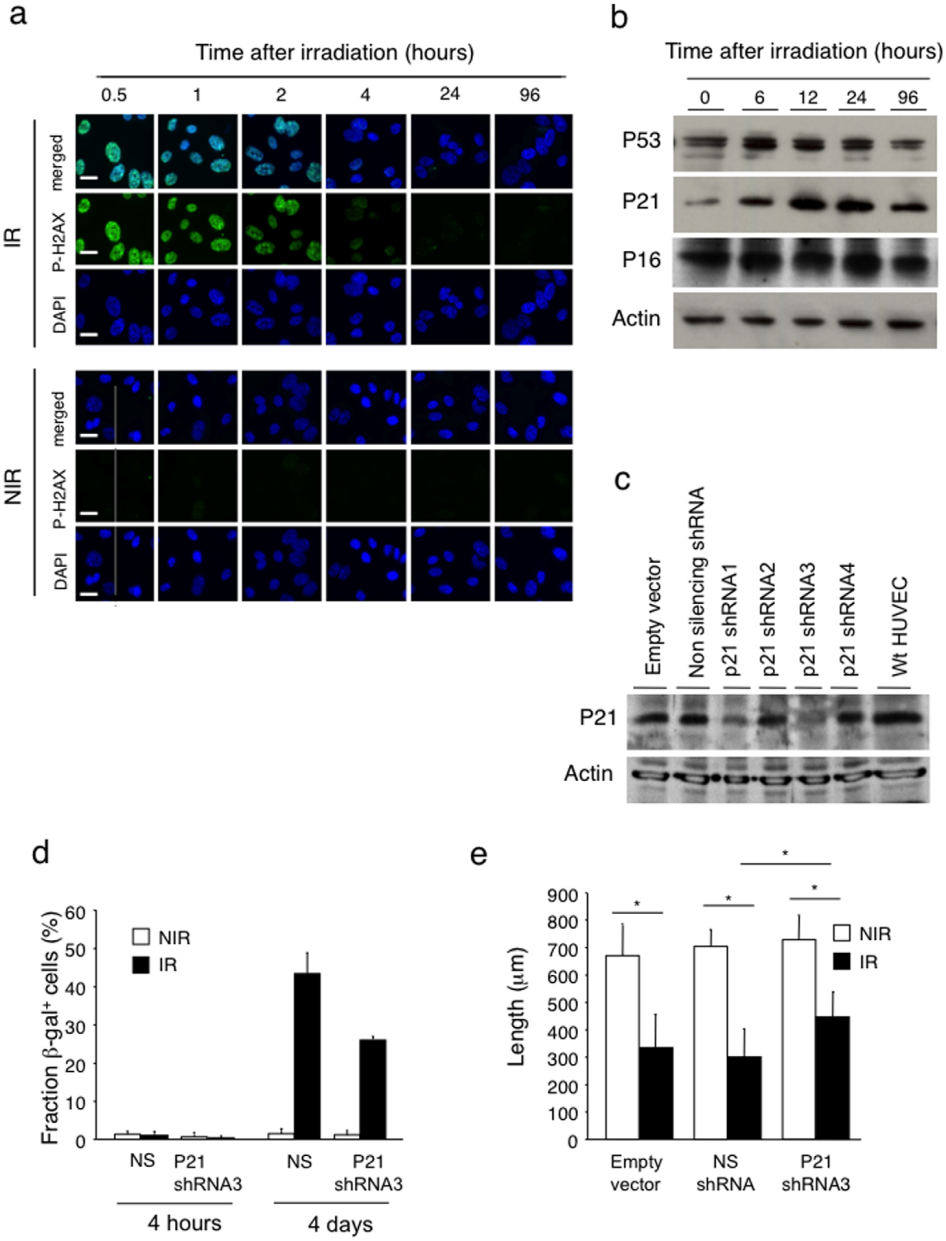
Role of radiation-induced activation of the P53-P21 pathway in senescence and migration. (a) Confluent HUVEC were irradiated at 15 Gy or not and analyzed for DNA double strand breaks by immunofluorescence staining using anti-P-H2AX mAb (green) at indicated time points. DAPI staining (blue) was used to detect nuclear DNA. Double strand breaks were repaired within 24 hours. (n = 5). Bars = 5 µm. (b) The expression level of three senescence-associated proteins, P16, P21 and P53, was analyzed by Western blotting before (0 hours) and after radiation at indicated time points. Actin was used as loading control. P53 levels are increased 6 hours after radiation, followed by an increase in P21 level. (n = 3). (c) HUVEC were transduced with lentiviruses expressing four different *P21*shRNA (1–4), no insert (empty vector) and non-silencing *shRNA*. HUVEC infected with *P21shRNA* # 3 were used for the functional experiments. (d) Senescence β-gal staining on control and P21 silenced HUVEC at 4 hours and 4 days after irradiation. (n = 6). (e) Migration ability of P21 silenced HUVEC was monitored by wound scratching assay. There was partial rescue of migration defect and decrease in number of senescence positive cells in P21 silenced HUVEC. (n = 3) *P<0.001. NIR: non-irradiated, IR: irradiated, NS: non-silencing HUVEC.

Two pathways are known to induce cell cycle arrest and senescence: the P16-Rb and P53-P21 pathways. Radiation is a potent inducer of P53 activation in response to double-strand DNA breaks [19]. Indeed, irradiation of confluent HUVEC induced DNA breaks as demonstrated by phospho (P)-H2AX nuclear staining, a hallmark of DNA double strand breaks [20] (Figure 7a). Twenty-four hours after irradiation P-H2AX returned to basal levels demonstrating effective DNA repair. Western blotting for P53, P21 and P16 proteins revealed a rapid enhancement of P53 protein followed by an increased in P21 levels, while P16 levels remained unchanged (Figure 7b). These results suggested the possible involvement of P21 in radiation-induced senescence and decreased sprouting, migration and proliferation. To test this hypothesis, we silenced P21 expression in HUVEC through lentivirus-mediated delivery of P21shRNA (Figure 7c). Silencing of P21 with the most efficient P21shRNA (shRNA3) partially rescued radiation-induced senescence (Figure 7d) and, to a minor extent, the migration defect (Figure 7e), but not the proliferation defect (Figure S3a).

To test for a role of P21 in radiation-induced inhibition of sprouting, we performed aortic ring sprouting assays using rings obtained from aorta of *p21*-null mice. Absence of p21 did not rescue radiation-induced inhibition of sprouting (15 Gy) compared to non-irradiated rings (Figure S3b). Consistent with this finding, aortic ring sprouting assays performed using aorta from *p53*-null mice did not rescue radiation-induced inhibition of sprouting (Figure S3c). We also performed sprouting assays using aortic rings obtained from *p16Ink4A* ^-/-^ mice. Lack of *p16* did not rescue the angiogenic defect of irradiated rings (data not shown), consistent with the absence of induction of P16 protein in irradiated HUVEC.

Taken together these results demonstrate that the P53-P21 pathway is induced upon irradiation in endothelial cells but appears not to be involved in suppressing endothelial sprouting, under the tested experimental conditions.

### Radiation activates the TGF-βRI/ALK5 pathway and ALK5 inhibition prevents radiation-induced suppression of migration and sprouting

**Figure 8:**
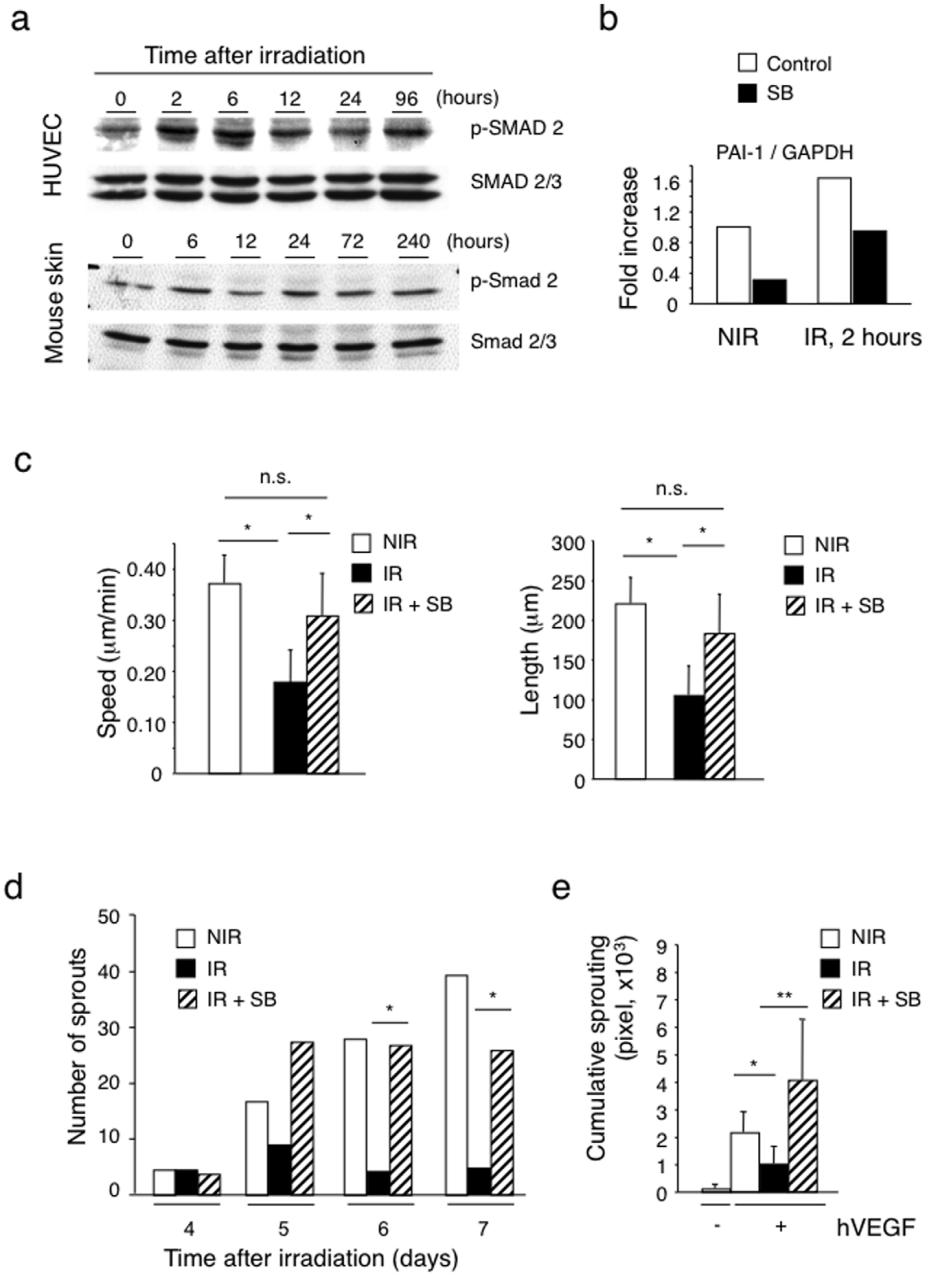
Radiation activates the TGF-βRI/ALK5 pathway and ALK5 inhibition prevents radiation-induced suppression of migration and sprouting. (a) Increased Smad2 phosphorylation in irradiated cultured HUVEC and mouse skin (15 Gy and 20 Gy respectively) demonstrated by Western blotting analysis. Induction of p-Smad2 was biphasic with early peaks at 2–6 hours and late peaks at 24–96 hours. (n = 3). (b) Radiation induces the TGF-β pathway target PAI-1 gene in HUVEC. HUVEC were treated with the ALK5 inhibitor SB431542 at 10 µM one day before radiation. RNA was extracted before and 2 hours after irradiation and PAI-1 mRNA quantified by real time RT-PCR. (c) The ALK5 inhibitor SB431542 (SB) rescued the migration defects caused by radiation. Left panel: migration speed; right panel: migration distance. (n = 10) *P<0.001. (d) The ALK5 inhibitor SB431542 (SB) rescued the sprouting defects caused by radiation. The SB compound was added in the medium during the whole mouse aortic ring assay. Level of endothelial cell sprouting in the presence of SB reached to levels observed in non-irradiated/non-treated rings (n = 9) *P<0.05. (e) The ALK5 inhibitor SB431542 (SB) rescued the radiation-induced sprouting defects in HUVEC spheroid assay. SB compound was added to spheroid-containing collagen gel at 10 µM and the spheroids were exposed to 15 Gy radiation. Endothelial sprouting was quantified 24 hours after incubation. (n = 10) *P<0.05, **P<0.001. NIR: non-irradiated, IR: irradiated, SB: SB431542.

Endothelial cells express two different TGF-β type I receptors, the activin receptor-like kinase (ALK) -1 and -5, which form heterodimers with TGF-β type II subunit. ALK-1 and ALK-5 have distinct activity profiles. ALK1 stimulates endothelial cell proliferation and migration via Smad1/5/8 transcription factors, whereas ALK5 inhibits endothelial cell proliferation and migration via Smad2/3 transcription factors [21]. Since TGF-β is induced by radiation *in vitro* and *in vivo* including in the skin [22], [23] and during tumor treatment with radio- and chemotherapy [24], we tested whether TGF-β signaling was activated in our model by monitoring Smad-2 phosphorylation in skin and HUVEC in response to ionizing radiation. Radiation induced a rapid increase in phospho-Smad-2 with an early peak at 6 hours followed by sustained phosphorylation (Figure 8a). Radiation also induced expression of *PAI-1* mRNA, an ALK5 target gene, which was inhibited by the small molecular ALK5 inhibitor SB431542 (Figure 8b). We then monitored the effect of radiation on *TGF-β1*, *ALK5* and *TGF-βRII* mRNA expression. *TGF-β1* and *ALK5* mRNA were expressed but not further induced by irradiation (Figure S4a and data not shown). In contrast, *TGF-βRII* mRNA expression was strongly induced by radiation with a biphasic profile (peak at 6 hours, over 20 fold induction, and increase again at 96 hours, ∼8 fold induction) (Figure S4b). Since TGF-β inhibits endothelial cell proliferation and migration through ALK5 [25], we tested the effect of the ALK5 inhibitor SB431542 on radiation-induced impairment of endothelial cell migration and sprouting. SB431542 treatment effectively prevented radiation-induced inhibition of migration of cultured HUVEC (Figure 8c) and restored sprouting in irradiated aortic rings (Figure 8d) and in irradiated HUVEC spheroids (Figure 8e) to levels observed under non-irradiated conditions. SB431542 treatment, however, did not rescue radiation-induced proliferation impairment (Figure S5a). Also, combined P21 silencing and SB431542 treatment did not prevent radiation-induced inhibition of proliferation (data not shown).

Ionizing radiation was recently reported to activate the Notch pathway in endothelial cells [26]. Since Notch signaling inhibits endothelial proliferation [27], sprouting [28] and angiogenesis [29], and TGF-β can activate Notch signaling [30] and cooperate with Notch activation in inhibiting cell proliferation [31], we tested whether the Notch pathway could be involved in impaired proliferation observed in our model. Indeed irradiation activated the Notch pathway, as demonstrated by the expression of the Notch target genes Hey1, and its inhibition by the γ-secretase inhibition GSI (Figure S5b). SB431542 did not inhibit radiation-induced Notch activation but it rather enhanced it (Figure S5b, black bar). GSI treatment, alone or in combination with SB431542 did not rescue radiation-induced suppression of endothelial cell proliferation (Figure S5c).

Taken together these results indicate that TGF-β/ALK5 signaling mediates radiation-induced inhibition of migration and sprouting, but not proliferation arrest, and exclude a contribution of radiation-induced Notch activation in proliferation arrest.

### ALK5 inhibition prevents radiation-induced inhibition of angiogenesis *in vivo*

**Figure 9:**
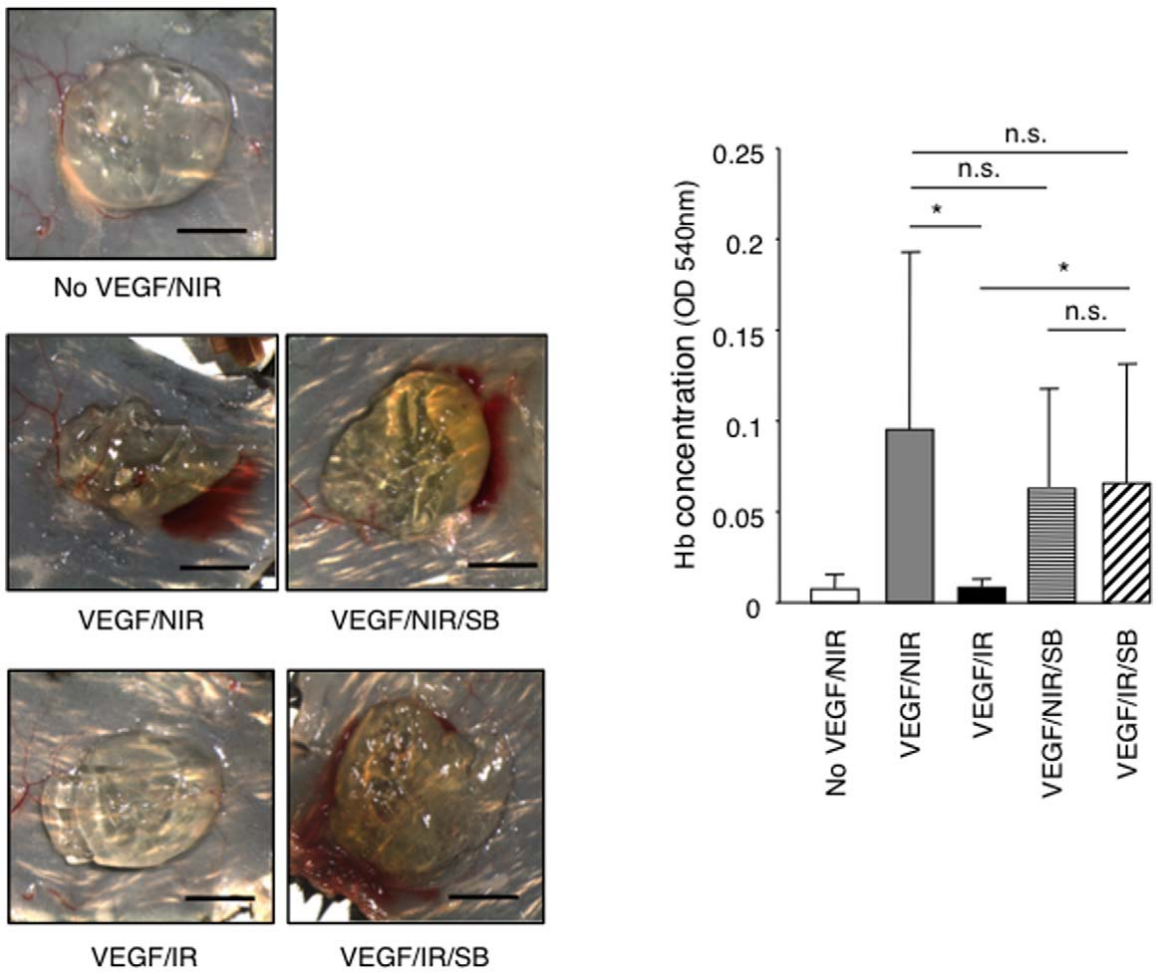
The ALK5 inhibitor SB431542 prevents radiation-induced inhibition of angiogenesis in the Matrigel plug assay. VEGF-induced angiogenesis was performed in non-irradiated or irradiated (20 Gy) C57/BL6 mice, which were treated with SB431542 (SB) compound, or vehicle only, daily from one day before irradiation until the end of the experiment. One week after Matrigel implantation, angiogenesis was quantified by measuring haemoglobin content. Representative images of recovered plugs and quantification of haemoglobin content are shown. (n = 7). Bars = 0.5 cm. *P<0.005. n.s., non significant. NIR: non-irradiated, IR: irradiated: SB: SB431542.

To test whether ALK5 inhibition was sufficient to restore angiogenesis *in vivo*, we performed a VEGF-driven Matrigel angiogenesis assays in non-irradiated mice and in locally pre-irradiated mice treated systemically with either the ALK5 inhibitor SB431542 or vehicle only. Treatment was started one day before irradiation and was pursued throughout the duration of the experiment. SB431542 treatment prevented the inhibition of VEGF-induced angiogenesis observed in pre-irradiated mice while it did not alter VEGF-induced angiogenesis in non-irradiated controls (Figure 9). Therefore we conclude that ALK5 signaling plays a dominant role in the suppression of angiogenesis induced by ionizing radiation *in vivo*.

## Discussion

Emerging evidence indicates that endothelial cells are sensitive to ionizing radiations, and endothelial dysfunctions induced by radiotherapy might be responsible for some of its wanted as well as unwanted effects. To date, however, no comprehensive characterization of such anti-angiogenic effect and its possible cellular and molecular mechanisms, have been reported. In this work we have investigated the effect of ionizing radiation on *de novo* angiogenesis using *in vivo, ex vivo* and *in vitro* experimental models in combination with genetic and pharmacological interventions. Here we report that: **i**) High doses of ionizing radiation suppressed angiogenesis *in vivo*, prevented endothelial cell re-entry into the cell cycle, inhibited migration and sprouting *in vitro* without causing increased apoptosis. **ii**) P53 and P21 were activated by radiation but their absence or silencing did not prevent radiation-induced inhibition of endothelial cell sprouting or proliferation. **iii**) Ionizing radiation induced ALK5-dependent signaling in endothelial cells and ALK5 inhibition rescued deficient endothelial cell sprouting and migration but not proliferation *in vitro*, and restored angiogenesis in irradiated mice *in vivo*.

A first important corollary of these observations is that ionizing radiation suppresses sprouting angiogenesis by acting directly on endothelial cells without the need for other cell types such as vessel wall cells or stromal cells. This conclusion is based on the cell autonomous inhibition of migration and sprouting in the spheroid assay. The critical endothelial cell events contributing to angiogenesis that are inhibited by radiation are migration and sprouting. In contrast, radiation-induced impaired proliferation and premature senescence seem not to be critical, at least within the tested experimental conditions. Radiation-induced endothelial cell senescence was reported earlier among irradiated (8 Gy) proliferating cells that survived irradiation after most cells died by apoptosis [32], [33]. Our results are consistent with these reports by showing that ionizing radiation kills proliferating endothelial cells while it induces senescence in quiescent cells. Since replicative senescent HUVEC also have reduced migratory capacities, one may consider that endothelial cell senescence, regardless of its cause, is associated with impaired migration. In contrast we could not obtain *in vivo* or *in vitro* experimental evidence for increased apoptosis of irradiated quiescent or confluent endothelial cells. While this negative result does not formally exclude that radiation-induced apoptosis may indeed occur, it nevertheless excludes apoptosis as the main mechanism of radiation-induced inhibition of *de novo* angiogenesis.

To unravel the molecular mechanisms possibly involved in mediating these radiation effects, we initially focused on the P53-P21 pathway for two reasons: first, this pathway is activated in irradiated endothelial cells, as a consequence of DNA double strand breaks and activation of DNA damage-sensing complexes [34] and, second, P21 expression causes cell cycle arrest and premature senescence, including in endothelial cells [35], [36]. Using aortic rings from mice deficient for *p53* or *p21* and silencing of P21 in HUVEC, however, we were unable to rescue proliferation arrest and deficient sprouting, and we could only partially prevent radiation-induced senescence and migration defects. Inhibition of stromal P53 through retrovirus-mediated delivery of dominant negative P53, was reported to enhance the anti-tumor angiogenesis effects of radiotherapy on tumoral vessels [37]. This, effect, however, is likely due to the enhancement of the genotoxic effect of radiotherapy on proliferating and retrovirally infected endothelial cells, and is therefore likely to be distinct from the effect reported here. The potential mechanism, by which P21 silencing partially prevented radiation-induced senescence and migration defect, but not sprouting or proliferation defects, is unclear at this point.

We found that radiation activates TGF-β type I receptor/ALK5 signaling and that inhibition of ALK5 kinase activity rescues deficient migration and sprouting *in vitro* and angiogenesis *in vivo*. These findings are consistent with and extend previously published observations on the induction of TGF-β by radiation and on its role in mediating radiation-induced biological effects [22], [38]. In the skin, TGF-β induction was reported as early as 6 hours after single dose of γ-irradiation [39], [40]. TGF-β was shown to inhibit cell proliferation through P21-depedent and independent mechanisms [41], [42], [43], [44] and to decrease cell migration by, at least in part, PAI-1 expression [44]. Further, constitutively active forms of ALK1 and ALK5 inhibited growth factor-induced endothelial sprouting from embryoid bodies [45]. In our model ALK5 inhibition, however, did not rescue the proliferation defect. We have also tested the role of Notch signaling in the inhibition of proliferation. Notch is activated by ionizing radiation [26] (and our data) and was shown to inhibit angiogenesis [46] and to induce endothelial cell proliferation arrest [47]. Notch inhibition, alone or in combination with ALK5 inhibition, however did not rescue the proliferation defect. In future experiments it will be interesting to inhibit TGF-β using blocking antibodies [24] to see whether the same rescue effect is observed.

The elusive nature of the mechanisms inhibiting proliferation and causing senescence is likely to reflect the complexity and redundancy of the mechanisms controlling such vital cellular functions as well as the multiplicity of signaling events elicited by ionizing radiations. Unraveling the mechanism of inhibited proliferation will require significant further work and may profit from a system biology approach [48].

The demonstration that inhibition of *de novo* angiogenesis is a direct effect of high-doses ionizing radiations on endothelial cells has two relevant implications to radiotherapy. On the one side it suggests that the curative-intent and adjuvant effects of radiotherapy may involve, at least in part, inhibition of tumor angiogenesis. The introduction of anti-angiogenic drugs into clinical practice has demonstrated that inhibition of tumor angiogenesis, mostly in combination with chemotherapy, extends progression-free and overall survival in several cancers [49]. Combination of anti-angiogenic drugs with radiotherapy may elicit even greater anti-tumor effects. At the same time, radiation-induced inhibition of *de novo* angiogenesis might also explain why cancers recurring within a previously irradiated field have a greater propensity to form metastases and are associated with poor prognosis compared to recurrences outside irradiated areas [50], [51], [52]. Others and we have shown that tumors growing within a preirradiated area have reduced angiogenesis and reduced growth, and are more hypoxic, more invasive and more metastatic compared to tumors growing in non-irradiated tissues [53], [54]. In those experiments, however, it was not clear whether inhibited angiogenesis was a primary effect of radiation or was a consequence of reduced tumor growth. Our findings support the first possibility thereby implicating inhibition of angiogenesis in the tumor bed effect. Chronic hypoxia of tumors growing within such an angiogenesis-deficient tissues will activate pro-invasive programs [55] or select for resistant cells with invasive characteristics [54]. This view is consistent with a recent report demonstrating that inhibition of tumor angiogenesis by anti-angiogenic drugs is associated with increased invasion and metastasis formation in experimental tumor models [56]. Such a mechanism, however, does not exclude that other ones might be involved in promoting post-radiation tumor invasion. In fact, TGF-β itself, a factor well-known to promote epithelial-mesenchymal transition [57], might also contribute to enhanced invasion and metastasis of cancers growing in a irradiated tissue [58]. These observations suggest the possibility of using ALK5 inhibitors during radiotherapy to prevent radiation-induced inhibition of angiogenesis and with it some of its unwanted effects. While this is an attractive option, great caution should be considered at this point since the TGF-β system has pleiotropic activities in normal tissue homeostasis and other pathological conditions and its imbalance may lead to unwanted or unpredictable effects on its own [59]. Inhibitors of ALK5 are in clinical development, including as anti-cancer agents [60] and results generated by these trials will be very informative on this matter.

This work has two intrinsic limitations. The first one is that, with one exception, we used single, high doses of radiation (8, 15 and 20 Gy), depending on the assay used. In contrast, clinical radiotherapy involves the repeated delivery of rather low doses of radiation, i.e. 1.8–2.2 Gy (fractionated therapy). While single high doses are often used in experimental models instead of fractionated therapy, caution should nevertheless be taken when translating results obtained with singles high doses into fractionated regimens used in the clinic. In fact we have little knowledge on how endothelial cells react to singles high doses of compared to multiple low doses [61]. Considering the clinical relevance of these effects, studies directly comparing the effects on tumor angiogenesis of fractionated *vs* single high-dose treatments are needed. The second limitation is that the effects on endothelial cells and angiogenesis were studies in the absence of a tumor. While this was done on purpose to selectively focus on direct radiation effects on quiescent endothelial cells, it is plausible that in the tumor microenvironment additional radiation-induced effects might come into play and overlap with the events reported here. A recent study demonstrated that tumor vessels re-growing after radiotherapy do so by switching from sprouting to intussusceptive angiogenesis, resulting in a 30% to 40% decrease in the intratumoral microvascular density at tumor recovery [62]. This switch in the mechanism of angiogenesis might explain the ability of tumors to growth in a pre-irradiated tissue in spite of fully blocked sprouting angiogenesis. Alternatively, radiation effects on bone marrow-derived cells may also contribute to the suppressed angiogenesis in irradiated tumors. Decreased matrix metalloproteinase-9 delivery by CD11b-positive bone marrow-derived myelomonocytic cells was shown to be responsible for the angiogenic blockade observed in irradiated tumors [63]. Furthermore, radiation was reported to mobilize bone marow-derived vascular progenitors [64] and CD11b^+^ cells which recruit to irradiated sites to promote vessel formation though a vasculogenic mechanism [65]. In future studies it will be important to compare radiation effects on endothelial cells *vs.* tumor-infiltrating bone marrow-derived cells in deficient angiogenesis and vasculogenic rescue.

In conclusion, our work demonstrates that radiation inhibits VEGF and FGF-2–induced angiogenesis through TGF-βRI/ALK5 –dependent (i.e. impaired migration and sprouting) and independent (i.e. cell cycle arrest and senescence) mechanisms. Radiation-induced endothelial cell apoptosis, and the p53-P21 pathway are not critically involved in radiation-induced suppression of angiogenesis. These results represent an important progress to the understanding of radiation-induced endothelial cell dysfunctions leading to impaired angiogenesis. Further studies are required to unravel additional mechanisms involved in these effects and to assess the clinical impact of these observations.

## Methods

### Antibodies and reagents

Primary antibodies for staining were purchased from the following companies; anti-CD31 (Pharmingen, San Diego, CA, USA), anti-αSMA cy3-conjugated (Sigma, Buchs, Switzerland), anti-phalloidin Alexa Fluor 568 conjugated (Molecular Probes, Eugene, OR, USA), anti-Paxillin (Transduction laboratories, Lexington, KY, USA), anti-phospho-H2AX (Upstate, Billerica, MA, USA), anti-P21, anti-P53, anti-P16, anti-Smad 2/3 and anti-phospho-Smad2 (Cell Signaling technology Inc. Danvers, MA, USA). Collagen I was from Upstate (Billerica, MA, USA). Recombinant human FGF-2 was from Peprotech EC Ltd (London, UK). Mouse VEGF was purchased from R&D Systems (Minneapolis, MN, USA). DAPI, Bovine serum albumin (BSA), paraformaldehyde (PFA), gelatin, fibronectin and collagen were purchased from Sigma Chemie (Buchs, Switzerland). Growth factor reduced Matrigel Matrix (MG) was from Becton Dickinson (Basel, Switzerland). TGF-β receptor I inhibitor: SB431542 was purchased from Tocris (Bristol, UK). The γ-secretase inhibitor DAPT was purchased from Calbiochem (San Diego, CA).

### Mice

C57/BL6 and Swiss nude mice were purchased from Harlan Nederland, Madison, WI, USA. p53 ^-/-^ mice were provided by Dr. Lawrence A. Donehower (Baylor College of Medicine, Houston, Texas, USA) and *p21* ^-/-^ and *p16Ink4A^-/-^* mice were provided by Dr. Friedrich Beermann (ISREC-EPFL, Lausanne, Switzerland). All experiments involving mice were performed in accordance with the guidelines of the Swiss Federal Veterinary Office (SFVO) and were approved by the Veterinary Office of Cantonal Vaud (Authorization number 1486.2).

### Cell lines and cell culture

HUVECs were prepared as previously described [66], and were cultured in 0.5% gelatin-coated dish in M199 medium (Invitrogen, Basel, Switzerland) supplemented with 10% FCS, 12 mg/ml of bovine brain extract (BBE: Clonetics, Walkersville, MD, USA), 10 ng/ml human recombinant EGF (Genzyme, Cambridge, MA, USA), 25 U/ml heparin, 1 µg/ml hydrocortisone (Sigma Chemie, Buchs, Switzerland) and 1% P/S. All cells were maintained in a humidified incubator at 37°C with 5% CO_2_.

### Irradiation

For *in vivo* irradiation experiments we used 6–10 weeks old C57/BL6 and Swiss nude female mice (Harlan Nederland, Madison, WI, USA). Before radiation, the mice were anesthetized with intra-peritoneal injection of ketamin/xylazine mixture. Local radiation was performed 7 days before Matrigel injection on the back with a single dose of 20 Gy by using a X-ray unit (PHILIPS, RT250, Germany), operated at 220 kV, 20 mA, with a 0.5 mm Cu filtration. The mice were placed into a lead jig allowing local irradiation sparing vital organs. The radiation field was 25×25 mm^2^, and a dose rate of 0.6 Gy/min was used. For whole body radiation, the mice were anesthetized as described above, and placed on the flat table. A single X-ray dose of 15 Gy or multiple doses of 3 Gy every 2 days until the same cumulated dose was reached (fractionated dose) were given to the mice using the same machine operated at 125 kV, 20 mA, with a Al filtration.

For *in vitro* radiation, a single dose of 8 Gy or 15 Gy was given to HUVEC and aortic rings.

### Matrigel plug angiogenesis assay

Matrigel plug angiogenesis assay was adapted from previously described assay [67]. Briefly, 7 days after 20 Gy local radiation of Swiss nude mice, two MG plug were (400 µl/plug) supplemented with FGF-2 (500 ng/ml) and Heparin (3 U/ml, Sigma Chemie, Buchs, Switzerland) and implanted within (dorsal) or outside (ventral) the irradiation area. For VEGF-induced angiogenesis, MG plugs was supplemented with mouse recombinant VEGF (200 ng/ml) and Heparin (10 U/ml) and implanted in C57/BL6 mice. C57/BL6 mice were used since VEGF-induced MG plug angiogenesis is more robust in these mice compared to Swiss nude mice. Seven days after MG injection, the plugs were removed and angiogenic responses were examined microscopically, biochemically and microscopically. Determination of hemoglobin content was performed using the Drabkin's reagent [68], or the diaminofluorene (DAF) method [69] (Sigma Chemie, Buchs Switzerland). Detection of CD31-positive endothelial cells was performed by immunostaining. Inhibition of ALK5 signaling was performed by the i.p. administration of SB431542 compound (10 mg/kg/mouse) daily starting one day before irradiation until the end of the experiment.

### Mouse aortic ring assay

The protocol of mouse aortic ring assay was kindly provided by Andrew Reynolds (Hodivala-Dilke's laboratory, London, UK). Eight weeks old adult C57/BL6 female mice were whole body irradiated at 15 Gy, and 5 days later they were sacrificed. The mice were dissected and the aorta was removed under sterile conditions in a laminar-flow hood using binocular microscope (Leica, MZ16, Heerbrugg, Switzerland). The aorta was cut in 1–2 mm thick rings, and all the rings were cultured in serum-free Optimem medium (Invitrogen, Basel, Switzerland) over night. The following day, collagen gels were prepared by using a 1.2 mg/ml collagen I solution (pH neutral) diluted in dH_2_O and mixed with 2xDMEM solution, and aliquoted into 50 µl/well in a 96 well tissue culture plate. The rings were then placed in the collagen solution at one ring per well. After 30 min polymerization of collagen at 37°C with 5% CO_2_, 150 µl of DMEM supplemented with 2.5% FCS and 30 ng/ml mouse recombinant VEGF were added over the collagen gel, and the plate was maintained in the incubator at 37°C and 5% CO_2_. The medium was refreshed each 2 days. ALK5 signaling was inhibited by adding SB431542 at 10 µM in the medium 2 days after embedding of rings in collagen gels. Sprouting angiogenesis was quantified by counting the number of branching sprouts. The sprouts were counted under the microscope between day 4 and 7 after initiation of the cultures and pictures were taken starting at the same time.

### Endothelial cell spheroid sprouting assay

To form spheroids, 1.6×10^5^ HUVEC were mixed with 1.2% methyl cellulose solution (Sigma, Buchs, Switzerland) and M119 complete medium. The cell suspension was distributed into 96-well U-shape culture plate (Greiner bio-one, Frickenhausen, Germany) and cultured overnight, following published protocols [16]. Next day, the spheroids were harvested and mixed with Rat tail collagen (pH neutral, final concentration at 2.4 mg/ml) diluted in dH_2_O, 2xDMEM, and 1.2% methyl cellulose solution, One ml of spheroids-containing collagen solution was aliquoted into wells in pre-warmed 24-well culture plates (Greiner bio-one) and the collagen was let to polymerize for 30 minutes at 37°C. 100 ml DMEM containing human VEGF (10 ng/ml final) and 10% FCS were added over the polymerized collagen gel, and immediately after the plate was irradiated with 15 Gy. Inhibition of ALK5 was achieved by adding the SB431542 compounds to the spheroids-containing gels at 10 µM final concentration. Twenty-four hours after incubation, endothelial sprouting was measured from 10 individual spheroids per condition.

### Cell survival and proliferation assay

Fully confluent HUVEC were exposed to 15 Gy radiation at single dose. Four days after the exposure, the cells were split at 1∶3 dilutions and further monitored for their growth for up to 5 days. The cells were cultured under normal condition at 37°C with 5% CO_2_. Non-irradiated HUVEC were used as control. SB431542 was added at 10 µM to the medium one day before the irradiation, and the medium was changed every 2 days throughout whole experimental period.

### Wound migration assay

Confluent HUVEC were prepared in 35 mm dishes and exposed to 15 Gy radiation at single dose. 4 days after radiation, a wound line was created in each dish. Floating cells were washed away with M199 medium. Cell migration was monitored for up to 20 hours at 6 minutes intervals by Time-lapse microscopy (Axiovert100 equipped with a heated CO_2_ chamber, Carl Zeiss, Feldbach, Switzerland). Images were analyzed with MetaMorph and ImageJ softwares. The speed and distance of migration of 10 single cells from one field was calculated for each condition with MTrackJ plugin in ImageJ. Three fields were chosen from each condition and data were analyzed by Student's *t*-test. The inhibition of ALK5 was performed by adding SB431542 at 10 µM in the medium 1 day before the radiation and the inhibitor containing medium was refreshed every 2 days.

### Cell adhesion assay

NUNC Maxisorp II (NUNC, Roskilde, Denmark) ELISA plates were coated with fibronectin (3 mg/ml) or collagen (1 mg/ml) over night at 4°C and blocked with 1% BSA for 1 hours at 37°C. Assays were done as previously described [66]. Briefly, Confluent irradiated HUVEC were maintained for 4 days after 15 Gy radiation, single dose. HUVEC were collected by trypsin digestion and seeded in serum-free M199 medium at 2×10^4^ cells/well. After the incubation at 37°C for 1 hour, the wells were gently washed with warm PBS and fixed for 30 minutes at RT with 2% paraformaldehyde (Fluka Chemie, Buchs, Switzerland). Cells were stained with 0.5% crystal violet, and absorbance of each well was read at 620 nm in a plate reader (Packard Spectra Count, Meriden, CT, USA). Results are normalized by BSA coated control wells and expressed as mean values of triplicate determinations ± s.d.

### Senescence-associated β-galactosidase assay

The senescence-associated β-galactosidase (β-gal) assay was performed using a senescence β-galactosidase staining kit (Cell signaling, Danvers, MA, USA) according to manufacturer's instructions. Confluent HUVEC were exposed to 15 Gy radiation. To generate aged senescence HUVEC, cultures were passaged for over 20 times at 1∶3 dilutions (approx 60 divisions). β-galactosidase-positive cells were counted under microscope (AXIOVERT 40CFL: Carl Zeiss, Feldbach, Switzerland) and normalized by the total number of cells. Values represent means ± s.d.

### Immunostainings

After radiation, mouse skin biopsies were taken at the indicated time points and frozen in OCT compound at −80°C. Explanted Matrigel plugs were frozen the same way. Eight mm thick sections were fixed in cold methanol for 10 minutes at 4°C. For CD31 immunohistochemical staining, sections were quenched in 0.3% H_2_O_2_ in water for 10 minutes. Thereafter, sections were blocked with 1.5% goat serum in PBS for 1 hour at room temperature (RT), and incubated with primary antibodies in the same buffer for 1 hour at RT or over night at 4°C. After washing, sections were incubated with the corresponding secondary antibodies labeled with either AlexaFluor488 or AlexaFluor546 (Molecular Probes, Eugene, OR, USA) for 1 hour at RT. Cell nuclei were counterstained with 20 ng/ml DAPI for 5 minutes at RT. For HUVEC staining, control or irradiated cultures were fixed with 2% PFA for 20 minutes at RT. Cells were permeabilized for 20 minutes at RT in 0.2% Triton X-100 in PBS, and then stained as above. First antibodies were used at following concentrations: anti-CD31 (1∶50), anti-paxillin (1∶50), anti-p-H2AX (1∶100). Actin was stained with Alexa Fluor 568-conjugated Phalloidin (1∶100 dilution), for 30 minutes at RT. TUNEL staining was performed using the In Situ Cell Death Detection Kit, Fluorescein (Roche, Basel, Switzerland) and following manufacturer's instructions.

### Flow cytometry

Confluent HUVEC were exposed to 15 Gy radiation and collected at 0, 6, 12, 24, and 96 hours after irradiation. The assay was performed according to manufacturer's instructions. Apoptosis was detected using the Annexin V-7AAD Apoptosis Detection Kit I (BD Biosciences, Allschwil, Switzerland) following manufacturer's instructions. For cell cycle analysis, confluent HUVEC were irradiated at 15 Gy, and 24 hours and 4 days later cells were harvested and fixed for 30 minutes at 4°C in FACS buffer containing 0.4% PFA. 1 ml FACS buffer containing 0.2% Triton-X-100 was added to the same tube and incubated over night at 4°C for permeabilization. Cells were stained with PE-Ki67 (BD Pharmingen, San Diego, CA, USA) for 1 hour at 4°C. After washing, 7-AAD (BD Pharmingen, San Diego, CA, USA) was added and cells incubated for 15 minutes at RT in the dark. Cells were analyzed using a FACScan II equipped for three color flow cytometry, and data were analyzed using CellQuest software (Becton Dickinson Biosciences, Mountain View, CA, USA).

### SDS-PAGE and Western blotting

Cells were lysed in ice cold modified RIPA buffer, sonicated and centrifuged at 13,000 rpm for 15 minutes at 4°C to remove debris. Proteins were resolved by SDS-PAGE and blotted onto Immobilon-P membranes (Milipore, Volketswil, Switzerland). Membranes were sequentially blocked in 5% dry milk for 1 hour at RT and then incubated with anti-p53, P21 and P16 antibodies at 1∶2000 dilution overnight at 4°C for primary antibody reaction. An HRP-labeled secondary antibody (DAKO, Zug, Switzerland) was used at 1∶2000 dilutions for 1 hour at RT. The ECL system was used for detection (Amersham-Pharmacia Biotech, Dübendorf, Switzerland). Unless otherwise indicated, experiments were carried out a minimum of three times to yield similar results. Representative experiments are shown.

### Real time RT-PCR

Gene expression was quantified by real time PCR on an ABI StepOne Plus machine (Applied Biosytems). Real-time PCR was carried out using SYBR Green master mix. The primer and Applied Biosystems gene expression essay used are given below. The comparative Ct method was used to calculate the difference of gene expression between samples. The 2-[DELTA][DELTA]Ct calculation is a convenient alternative method to the relative Standard Curve Method to derive accurate quantitative information from real time PCR assays [70]. Quantity of cDNA was normalized using the housekeeping gene GAPDH. The following primers were used:

human GAPDH forward (F): 5′-ATCCCATCACCATCTTCCAG-3′; human GAPDH reverse (R): 5′- CGAAATCCCAAACTCCGATAGTC-3′; human PAI-1 F: 5′-CATCCCCCATCCTACGTGG-3′; human PAI-1 R: 5′-CCCCATAGGGTGAGAAAACCA-3′; human TGF-β1 forward (F): GCAACAATTCCTGGCGATAC; human TGF-β1 reverse (R): GAACCCGTTGATGTCCACTT; human TGF-βRII forward (F): TTCAAGTGACAGGCATCAGC; human TGF-βRII reverse (R): GGTTGATGTTGTTGGCACAC

### Gene silencing

For P21 silencing in HUVEC, four different clones of *p21* shRNA lentivirus particles were purchased from OpenBiosystemes (RHS4531, NM_078467: Huntsville, USA). HUVEC were transduced with each one of the lentivirus particles (MOI = 8) following the supplied protocol. Forty-eight hours later, transduced cells were selected by culturing them for 3 days in complete medium supplemented with 3 µg/ml puromycine followed by expansion in medium alone. Suppressed P21 expression was demonstrated by Western blotting. *p21shRNA3* gave the best silencing efficiency and was used in functional experiments.

### Statistical Analysis

Data were analyzed by Student's *t*-test for both *in vitro* and *in vivo* experiments using the statistical package in MS Excel Version 11.5.5 for Mac (Microsoft Corporation, Redmond, OR). *P* values <0.05 were considered significant. Results are expressed as mean ± s.d. depicted by error bars (95% confidence interval).

## Supporting Information

Figure S1 Ionizing radiation kills proliferating but not quiescent HUVEC in vitro. (a) Confluent HUVEC were exposed to 15 Gy (IR) X-ray radiation and monitored for changes in morphology and survival before irradiation (0 hours) and at 24 hours and 4 days after irradiation, and compared to non-irradiated (NIR) HUVEC. No cell loss and no detectable morphological or density differences between IR and NIR HUVEC were observed. Bars = 30 µm. (b) Sub-confluent (50%) HUVEC were irradiated at 15 Gy (IR) and observed before and at 24 hours and 4 days after irradiation and compared to non-irradiated (NIR) HUVEC. Irradiated sub-confluent HUVEC massively died within 4 days after irradiation, whereas non-irradiated cultures proliferated and reached confluence. Bars = 30 µm.
(3.25 MB TIF)

Figure S2 Replicative senescence of HUVEC is associated with reduced migration. Replicative senescent HUVEC cells (RS) were prepared by passaging cultures 20 times. The migration capacities of non-irradiated HUVEC (NIR), replicative senescent HUVEC and 15 Gy irradiated HUVEC (IR) were tested by the scratch wound closure assay in which individual cells were monitored for their migration speed. RS and IR HUVEC have reduced migratory capacities.
(3.25 MB TIF)

Figure S3 P53 or P21 deficiency does not prevent radiation-induced proliferation arrest or sprouting (a) Confluent, non-silenced (NS) and P21 silenced (P21shRNA3) HUVEC were irradiated at 15 Gy, and 4 days later they were split at 1∶3 dilutions to monitor their proliferation ability. P21 silencing dramatically increased proliferation of non-irradiated HUVEC, but did not rescue the proliferation defect in irradiated HUVEC. (b) Mouse aortic ring assay from p21 null mice. The mice were exposed to 15 Gy whole body irradiation 5 days before the aorta was dissected. p21 deficiency did not rescue the radiation-induced inhibition of sprouting. *P<0.05, **P<0.01. (c) Mouse aortic ring assay from p53 null mice. Wild type and p53 null mice were exposed to 15 Gy whole body radiation 5 days before aorta dissection. Absence of p53 did not rescue the inhibition of sprouting by ionizing radiation. *P<0.01. NIR: non-irradiated, IR: irradiated, NS: non-silencing.
(3.25 MB TIF)

Figure S4 Radiation induces TGFβRII, but not TGFβ, mRNA expression in endothelial cells. (a) Endothelial cells were irradiated with 15 Gy single dose, total RNA was extracted before (t = 0) and at 6, 12, 24 and 96 hours after irradiation and TGFβ mRNA quantified by real time RT-PCR. (b) Endothelial cells were irradiated with 15 Gy single dose, total RNA was extracted before (t = 0) and at 2, 6, 12, 24 and 96 hours after irradiation and TGFβRII mRNA quantified by real time RT-PCR. NIR: non-irradiated, IR: irradiated. Representatives of duplicate experiments are shown.
(3.25 MB TIF)

Figure S5 Inhibition of ALK5, alone of in combination with Notch inhibition, does not rescue radiation-induced proliferation arrest. (a) HUVEC were treated with the ALK5 inhibitor SB431542 at 10 µM, 24 hours before radiation. Four days after radiation, the cells were split at 1∶3 dilutions to monitor further proliferation. In the presence of SB431542 non-irradiated HUVEC significantly increased their proliferation, however there was no rescue of proliferation defect of irradiated cells. *P<0.001. (b) HUVEC were treated with the ALK5 inhibitor SB431542 and the γ-secretase inhibitor GSI at 10 µM one day before radiation. RNA was extracted from non-irradiated HUVEC and from HUVEC 2 hours after irradiation, and Hey1 mRNA expression analyzed by real time RT-PCR. Radiation induced Hey-1, which was blocked by GSI or GSI+SB, but was enhanced by SB alone. (c) Effect of GSI, SB431542, singly and in combination, on inhibition of HUVEC proliferation following radiation. Inhibitors were added in the medium 1 day before radiation. HUVEC were exposed to 15 Gy radiation and cultured for 4 days. Cells were split at 1∶3 dilutions and the cell proliferation was monitored at 1, 2, 3 and 4 days after splitting. There was no rescue of radiation-induced proliferation defects by blocking Notch alone or in combination with ALK5 inhibition. NIR: non-irradiated, IR: irradiated, SB: SB431542, GSI, γ-secretase inhibitor.
(3.25 MB TIF)

## References

[1] Bernier J, Hall EJ, Giaccia A (2004). Radiation oncology: a century of achievements.. Nat Rev Cancer.

[2] Rosen EM, Fan S, Rockwell S, Goldberg ID (1999). The molecular and cellular basis of radiosensitivity: implications for understanding how normal tissues and tumors respond to therapeutic radiation.. Cancer Invest.

[3] Barcellos-Hoff MH, Park C, Wright EG (2005). Radiation and the microenvironment - tumorigenesis and therapy.. Nat Rev Cancer.

[4] Garcia-Barros M, Paris F, Cordon-Cardo C, Lyden D, Rafii S (2003). Tumor response to radiotherapy regulated by endothelial cell apoptosis.. Science.

[5] Fajardo LF (2005). The pathology of ionizing radiation as defined by morphologic patterns.. Acta Oncol.

[6] Tetik O, Yetkin U, Calli AO, Ilhan G, Gurbuz A (2008). Occlusive arterial disease after radiotherapy for testicular cancer: case report and review of the literature.. Vascular.

[7] Group EBCTC (2000). Favourable and unfavourable effects on long-term survival of radiotherapy for early breast cancer: an overview of the randomised trials.. Lancet.

[8] Dorresteijn LD, Kappelle AC, Scholz NM, Munneke M, Scholma JT (2005). Increased carotid wall thickening after radiotherapy on the neck.. Eur J Cancer.

[9] Dormand EL, Banwell PE, Goodacre TE (2005). Radiotherapy and wound healing.. Int Wound J.

[10] Akhtar N, Dickerson EB, Auerbach R (2002). The sponge/Matrigel angiogenesis assay.. Angiogenesis.

[11] Barton M (1995). Tables of equivalent dose in 2 Gy fractions: a simple application of the linear quadratic formula.. Int J Radiat Oncol Biol Phys.

[12] Lyng FM, Maguire P, Kilmurray N, Mothersill C, Shao C (2006). Apoptosis is initiated in human keratinocytes exposed to signalling factors from microbeam irradiated cells.. Int J Radiat Biol.

[13] Qu J, Cheng T, Shi C, Lin Y, Ran X (2004). A study on the activity of fibroblast cells in connection with tissue recovery in the wounds of skin injury after whole-body irradiation.. J Radiat Res (Tokyo).

[14] Davis TA, Mungunsukh O, Zins S, Day RM, Landauer MR (2008). Genistein induces radioprotection by hematopoietic stem cell quiescence.. Int J Radiat Biol.

[15] Nicosia RF, Ottinetti A (1990). Growth of microvessels in serum-free matrix culture of rat aorta. A quantitative assay of angiogenesis in vitro.. Lab Invest.

[16] Korff T, Augustin HG (1998). Integration of endothelial cells in multicellular spheroids prevents apoptosis and induces differentiation.. J Cell Biol.

[17] Blacher S, Devy L, Burbridge MF, Roland G, Tucker G (2001). Improved quantification of angiogenesis in the rat aortic ring assay.. Angiogenesis.

[18] Munoz-Chapuli R, Quesada AR, Angel Medina M (2004). Angiogenesis and signal transduction in endothelial cells.. Cell Mol Life Sci.

[19] Fei P, El-Deiry WS (2003). P53 and radiation responses.. Oncogene.

[20] Kuo LJ, Yang LX (2008). Gamma-H2AX - a novel biomarker for DNA double-strand breaks.. In Vivo.

[21] Lebrin F, Deckers M, Bertolino P, Ten Dijke P (2005). TGF-beta receptor function in the endothelium.. Cardiovasc Res.

[22] Barcellos-Hoff MH, Derynck R, Tsang ML, Weatherbee JA (1994). Transforming growth factor-beta activation in irradiated murine mammary gland.. J Clin Invest.

[23] Randall K, Coggle JE (1996). Long-term expression of transforming growth factor TGF beta 1 in mouse skin after localized beta-irradiation.. Int J Radiat Biol.

[24] Biswas S, Guix M, Rinehart C, Dugger TC, Chytil A (2007). Inhibition of TGF-beta with neutralizing antibodies prevents radiation-induced acceleration of metastatic cancer progression.. J Clin Invest.

[25] Castanares C, Redondo-Horcajo M, Magan-Marchal N, ten Dijke P, Lamas S (2007). Signaling by ALK5 mediates TGF-beta-induced ET-1 expression in endothelial cells: a role for migration and proliferation.. J Cell Sci.

[26] Scharpfenecker M, Kruse JJ, Sprong D, Russell NS, Ten Dijke P (2009). Ionizing radiation shifts the PAI-1/ID-1 balance and activates notch signaling in endothelial cells.. Int J Radiat Oncol Biol Phys.

[27] Liu ZJ, Xiao M, Balint K, Soma A, Pinnix CC (2006). Inhibition of endothelial cell proliferation by Notch1 signaling is mediated by repressing MAPK and PI3K/Akt pathways and requires MAML1.. FASEB J.

[28] Siekmann AF, Lawson ND (2007). Notch signalling limits angiogenic cell behaviour in developing zebrafish arteries.. Nature.

[29] Takeshita K, Satoh M, Ii M, Silver M, Limbourg FP (2007). Critical role of endothelial Notch1 signaling in postnatal angiogenesis.. Circ Res.

[30] Zavadil J, Cermak L, Soto-Nieves N, Bottinger EP (2004). Integration of TGF-beta/Smad and Jagged1/Notch signalling in epithelial-to-mesenchymal transition.. EMBO J.

[31] Niimi H, Pardali K, Vanlandewijck M, Heldin CH, Moustakas A (2007). Notch signaling is necessary for epithelial growth arrest by TGF-beta.. J Cell Biol.

[32] Igarashi K, Sakimoto I, Kataoka K, Ohta K, Miura M (2007). Radiation-induced senescence-like phenotype in proliferating and plateau-phase vascular endothelial cells.. Exp Cell Res.

[33] Oh CW, Bump EA, Kim JS, Janigro D, Mayberg MR (2001). Induction of a senescence-like phenotype in bovine aortic endothelial cells by ionizing radiation.. Radiat Res.

[34] Lakin ND, Jackson SP (1999). Regulation of p53 in response to DNA damage.. Oncogene.

[35] Miyauchi H, Minamino T, Tateno K, Kunieda T, Toko H (2004). Akt negatively regulates the in vitro lifespan of human endothelial cells via a p53/p21-dependent pathway.. EMBO J.

[36] Spyridopoulos I, Isner JM, Losordo DW (2002). Oncogenic ras induces premature senescence in endothelial cells: role of p21(Cip1/Waf1).. Basic Res Cardiol.

[37] Burdelya LG, Komarova EA, Hill JE, Browder T, Tararova ND (2006). Inhibition of p53 response in tumor stroma improves efficacy of anticancer treatment by increasing antiangiogenic effects of chemotherapy and radiotherapy in mice.. Cancer Res.

[38] Barcellos-Hoff MH (1993). Radiation-induced transforming growth factor beta and subsequent extracellular matrix reorganization in murine mammary gland.. Cancer Res.

[39] Martin M, Vozenin MC, Gault N, Crechet F, Pfarr CM (1997). Coactivation of AP-1 activity and TGF-beta1 gene expression in the stress response of normal skin cells to ionizing radiation.. Oncogene.

[40] Randall K, Coggle JE (1995). Expression of transforming growth factor-beta 1 in mouse skin during the acute phase of radiation damage.. Int J Radiat Biol.

[41] Datto MB, Li Y, Panus JF, Howe DJ, Xiong Y (1995). Transforming growth factor beta induces the cyclin-dependent kinase inhibitor p21 through a p53-independent mechanism.. Proc Natl Acad Sci U S A.

[42] Hannon GJ, Beach D (1994). p15INK4B is a potential effector of TGF-beta-induced cell cycle arrest.. Nature.

[43] Koff A, Ohtsuki M, Polyak K, Roberts JM, Massague J (1993). Negative regulation of G1 in mammalian cells: inhibition of cyclin E-dependent kinase by TGF-beta.. Science.

[44] Goumans MJ, Valdimarsdottir G, Itoh S, Rosendahl A, Sideras P (2002). Balancing the activation state of the endothelium via two distinct TGF-beta type I receptors.. Embo J.

[45] Mallet C, Vittet D, Feige JJ, Bailly S (2006). TGFbeta1 induces vasculogenesis and inhibits angiogenic sprouting in an embryonic stem cell differentiation model: respective contribution of ALK1 and ALK5.. Stem Cells.

[46] Leong KG, Hu X, Li L, Noseda M, Larrivee B (2002). Activated Notch4 inhibits angiogenesis: role of beta 1-integrin activation.. Mol Cell Biol.

[47] Noseda M, Chang L, McLean G, Grim JE, Clurman BE (2004). Notch activation induces endothelial cell cycle arrest and participates in contact inhibition: role of p21Cip1 repression.. Mol Cell Biol.

[48] Barcellos-Hoff MH, Costes SV (2006). A systems biology approach to multicellular and multi-generational radiation responses.. Mutat Res.

[49] Kerbel RS (2008). Tumor angiogenesis.. N Engl J Med.

[50] Vicini FA, Kestin L, Huang R, Martinez A (2003). Does local recurrence affect the rate of distant metastases and survival in patients with early-stage breast carcinoma treated with breast-conserving therapy?. Cancer.

[51] Vikram B, Strong EW, Shah JP, Spiro R (1984). Failure at distant sites following multimodality treatment for advanced head and neck cancer.. Head Neck Surg.

[52] Suit HD (1992). Local control and patient survival.. Int J Radiat Oncol Biol Phys.

[53] Rofstad EK, Mathiesen B, Henriksen K, Kindem K, Galappathi K (2005). The tumor bed effect: increased metastatic dissemination from hypoxia-induced up-regulation of metastasis-promoting gene products.. Cancer Res.

[54] Monnier Y, Farmer P, Bieler G, Imaizumi N, Sengstag T (2008). CYR61 and alphaVbeta5 integrin cooperate to promote invasion and metastasis of tumors growing in preirradiated stroma.. Cancer Res.

[55] Chan DA, Giaccia AJ (2007). Hypoxia, gene expression, and metastasis.. Cancer Metastasis Rev.

[56] Paez-Ribes M, Allen E, Hudock J, Takeda T, Okuyama H (2009). Antiangiogenic therapy elicits malignant progression of tumors to increased local invasion and distant metastasis.. Cancer Cell.

[57] Jakowlew SB (2006). Transforming growth factor-beta in cancer and metastasis.. Cancer Metastasis Rev.

[58] Park CC, Bissell MJ, Barcellos-Hoff MH (2000). The influence of the microenvironment on the malignant phenotype.. Mol Med Today.

[59] Kim IY, Kim MM, Kim SJ (2005). Transforming growth factor-beta: biology and clinical relevance.. J Biochem Mol Biol.

[60] Lahn M, Kloeker S, Berry BS (2005). TGF-beta inhibitors for the treatment of cancer.. Expert Opin Investig Drugs.

[61] Brown JM, Koong AC (2008). High-dose single-fraction radiotherapy: exploiting a new biology?. Int J Radiat Oncol Biol Phys.

[62] Hlushchuk R, Riesterer O, Baum O, Wood J, Gruber G (2008). Tumor recovery by angiogenic switch from sprouting to intussusceptive angiogenesis after treatment with PTK787/ZK222584 or ionizing radiation.. Am J Pathol.

[63] Ahn GO, Brown JM (2008). Matrix metalloproteinase-9 is required for tumor vasculogenesis but not for angiogenesis: role of bone marrow-derived myelomonocytic cells.. Cancer Cell.

[64] Allan DS, Morgan SC, Birch PE, Yang L, Halpenny MJ (2009). Mobilization of circulating vascular progenitors in cancer patients receiving external beam radiation in response to tissue injury.. Int J Radiat Oncol Biol Phys.

[65] Kioi M, Vogel H, Schultz G, Hoffman RM, Harsh GR (2010). Inhibition of vasculogenesis, but not angiogenesis, prevents the recurrence of glioblastoma after irradiation in mice.. J Clin Invest.

[66] Ruegg C, Yilmaz A, Bieler G, Bamat J, Chaubert P (1998). Evidence for the involvement of endothelial cell integrin alphaVbeta3 in the disruption of the tumor vasculature induced by TNF and IFN-gamma.. Nat Med.

[67] Passaniti A, Taylor RM, Pili R, Guo Y, Long PV (1992). A simple, quantitative method for assessing angiogenesis and antiangiogenic agents using reconstituted basement membrane, heparin, and fibroblast growth factor.. Lab Invest.

[68] Balasubramaniam P, Malathi A (1992). Comparative study of hemoglobin estimated by Drabkin's and Sahli's methods.. J Postgrad Med.

[69] Worthington RE, Bossie-Codreanu J, Van Zant G (1987). Quantitation of erythroid differentiation in vitro using a sensitive colorimetric assay for hemoglobin.. Exp Hematol.

[70] Arocho A, Chen B, Ladanyi M, Pan Q (2006). Validation of the 2-DeltaDeltaCt calculation as an alternate method of data analysis for quantitative PCR of BCR-ABL P210 transcripts.. Diagn Mol Pathol.

