# Supplementary figures and images for: Radiotherapy Suppresses Angiogenesis in Mice through TGF-βRI/ALK5-Dependent Inhibition of Endothelial Cell Sprouting
Source: PLoS One. 2010 Jun 11;5(6):e11084. doi: 10.1371/journal.pone.0011084 (PMC2884035; doi:10.1371/journal.pone.0011084)

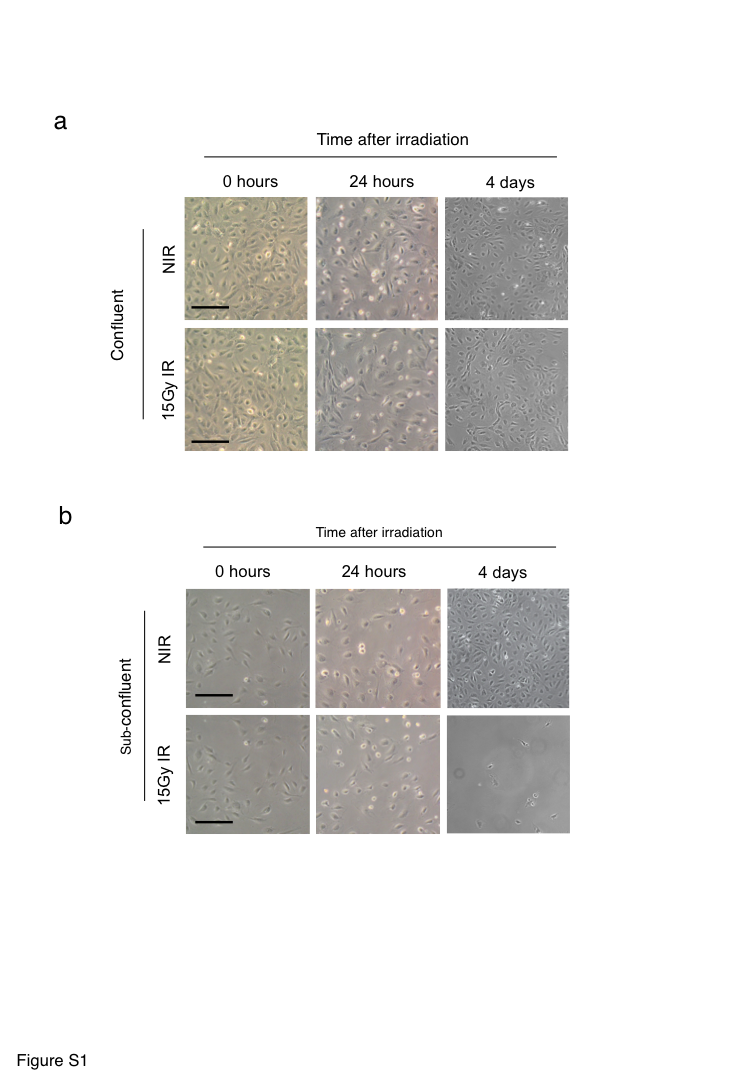

Supplement: Figure S1 — Ionizing radiation kills proliferating but not quiescent HUVEC in vitro. (a) Confluent HUVEC were exposed to 15 Gy (IR) X-ray radiation and monitored for changes in morphology and survival before irradiation (0 hours) and at 24 hours and 4 days after irradiation, and compared to non-irradiated (NIR) HUVEC. No cell loss and no detectable morphological or density differences between IR and NIR HUVEC were observed. Bars = 30 µm. (b) Sub-confluent (50%) HUVEC were irradiated at 15 Gy (IR) and observed before and at 24 hours and 4 days after irradiation and compared to non-irradiated (NIR) HUVEC. Irradiated sub-confluent HUVEC massively died within 4 days after irradiation, whereas non-irradiated cultures proliferated and reached confluence. Bars = 30 µm. (3.25 MB TIF) [file pone.0011084.s001.tif]

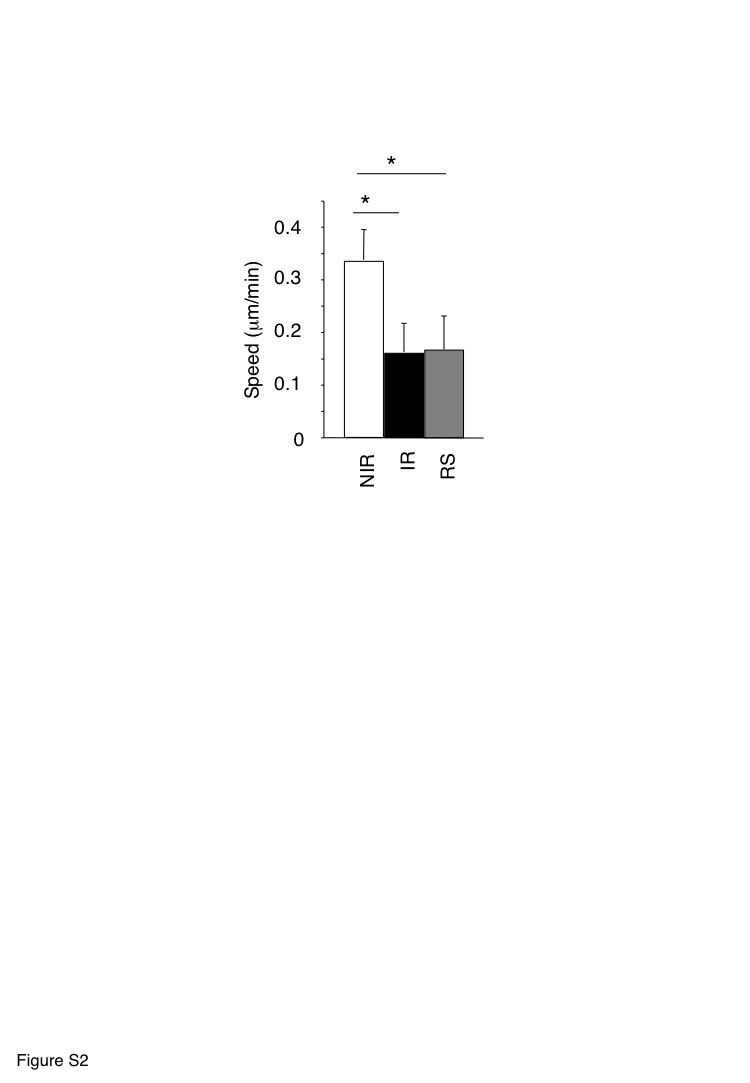

Supplement: Figure S2 — Replicative senescence of HUVEC is associated with reduced migration. Replicative senescent HUVEC cells (RS) were prepared by passaging cultures 20 times. The migration capacities of non-irradiated HUVEC (NIR), replicative senescent HUVEC and 15 Gy irradiated HUVEC (IR) were tested by the scratch wound closure assay in which individual cells were monitored for their migration speed. RS and IR HUVEC have reduced migratory capacities. (3.25 MB TIF) [file pone.0011084.s002.tif]

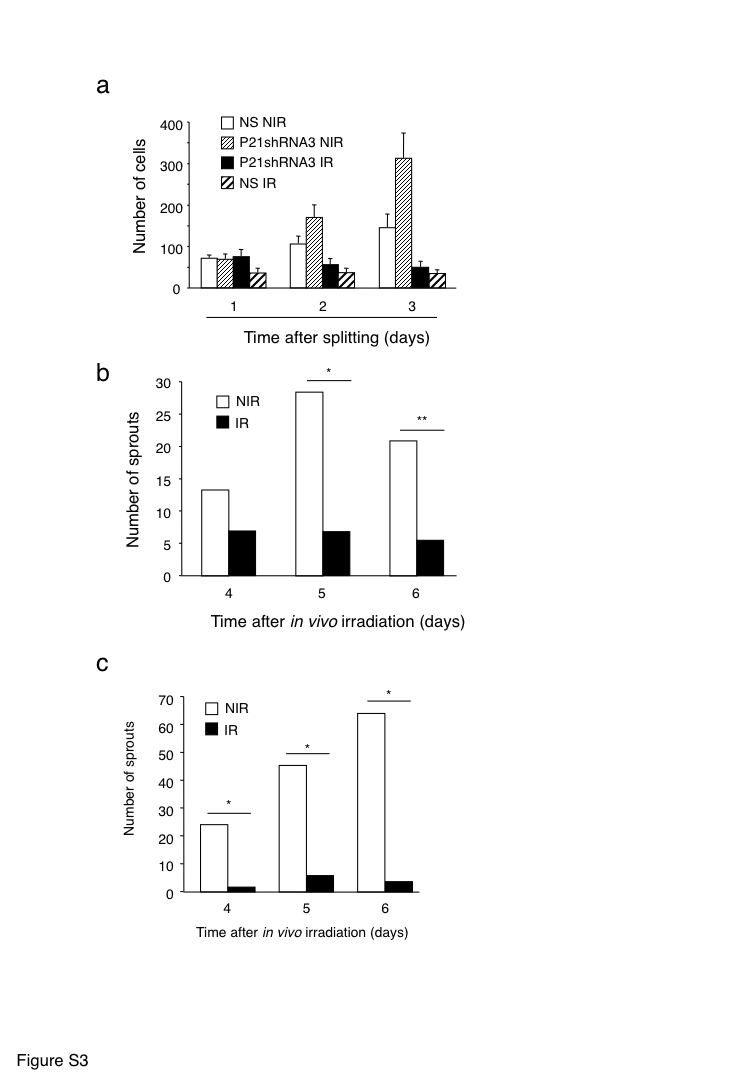

Supplement: Figure S3 — P53 or P21 deficiency does not prevent radiation-induced proliferation arrest or sprouting (a) Confluent, non-silenced (NS) and P21 silenced (P21shRNA3) HUVEC were irradiated at 15 Gy, and 4 days later they were split at 1∶3 dilutions to monitor their proliferation ability. P21 silencing dramatically increased proliferation of non-irradiated HUVEC, but did not rescue the proliferation defect in irradiated HUVEC. (b) Mouse aortic ring assay from p21 null mice. The mice were exposed to 15 Gy whole body irradiation 5 days before the aorta was dissected. p21 deficiency did not rescue the radiation-induced inhibition of sprouting. *P<0.05, **P<0.01. (c) Mouse aortic ring assay from p53 null mice. Wild type and p53 null mice were exposed to 15 Gy whole body radiation 5 days before aorta dissection. Absence of p53 did not rescue the inhibition of sprouting by ionizing radiation. *P<0.01. NIR: non-irradiated, IR: irradiated, NS: non-silencing. (3.25 MB TIF) [file pone.0011084.s003.tif]

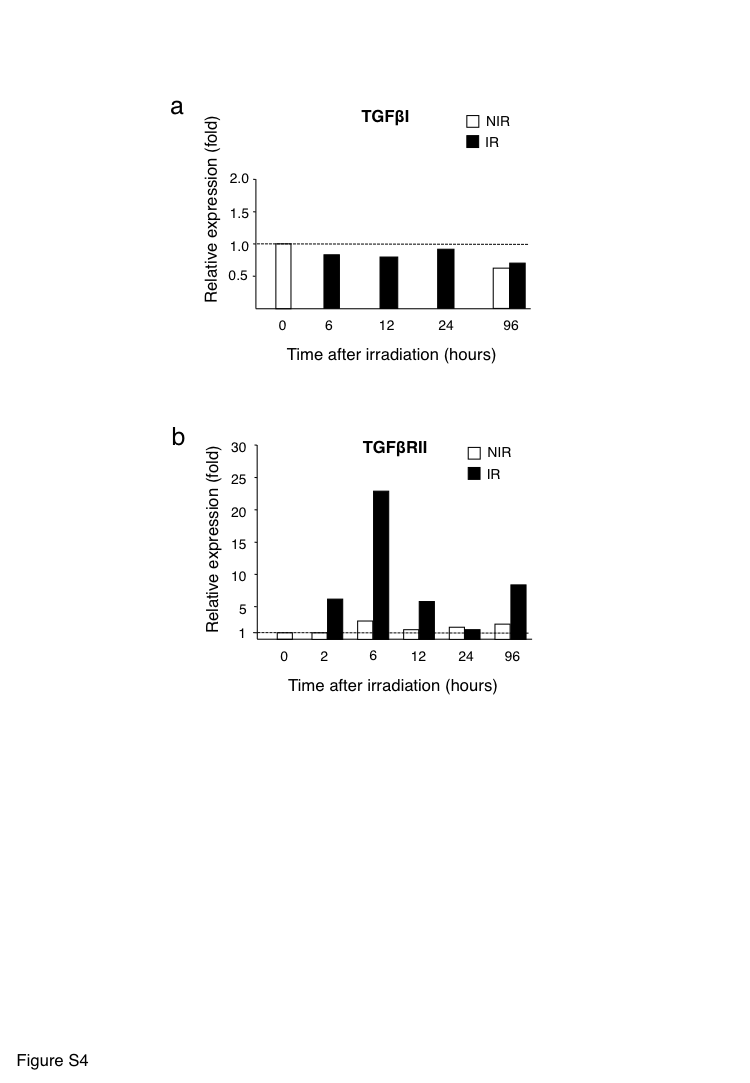

Supplement: Figure S4 — Radiation induces TGFβRII, but not TGFβ, mRNA expression in endothelial cells. (a) Endothelial cells were irradiated with 15 Gy single dose, total RNA was extracted before (t = 0) and at 6, 12, 24 and 96 hours after irradiation and TGFβ mRNA quantified by real time RT-PCR. (b) Endothelial cells were irradiated with 15 Gy single dose, total RNA was extracted before (t = 0) and at 2, 6, 12, 24 and 96 hours after irradiation and TGFβRII mRNA quantified by real time RT-PCR. NIR: non-irradiated, IR: irradiated. Representatives of duplicate experiments are shown. (3.25 MB TIF) [file pone.0011084.s004.tif]

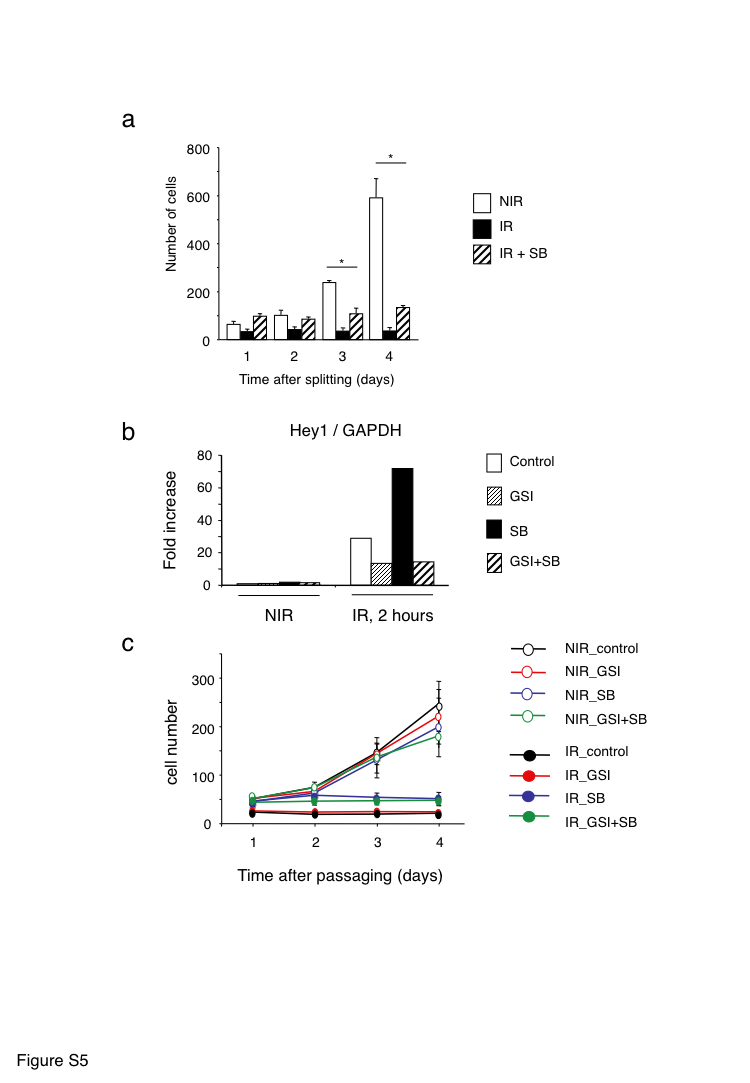

Supplement: Figure S5 — Inhibition of ALK5, alone of in combination with Notch inhibition, does not rescue radiation-induced proliferation arrest. (a) HUVEC were treated with the ALK5 inhibitor SB431542 at 10 µM, 24 hours before radiation. Four days after radiation, the cells were split at 1∶3 dilutions to monitor further proliferation. In the presence of SB431542 non-irradiated HUVEC significantly increased their proliferation, however there was no rescue of proliferation defect of irradiated cells. *P<0.001. (b) HUVEC were treated with the ALK5 inhibitor SB431542 and the γ-secretase inhibitor GSI at 10 µM one day before radiation. RNA was extracted from non-irradiated HUVEC and from HUVEC 2 hours after irradiation, and Hey1 mRNA expression analyzed by real time RT-PCR. Radiation induced Hey-1, which was blocked by GSI or GSI+SB, but was enhanced by SB alone. (c) Effect of GSI, SB431542, singly and in combination, on inhibition of HUVEC proliferation following radiation. Inhibitors were added in the medium 1 day before radiation. HUVEC were exposed to 15 Gy radiation and cultured for 4 days. Cells were split at 1∶3 dilutions and the cell proliferation was monitored at 1, 2, 3 and 4 days after splitting. There was no rescue of radiation-induced proliferation defects by blocking Notch alone or in combination with ALK5 inhibition. NIR: non-irradiated, IR: irradiated, SB: SB431542, GSI, γ-secretase inhibitor. (3.25 MB TIF) [file pone.0011084.s005.tif]
